# Maximum electromagnetic local density of states via material structuring

**DOI:** 10.1515/nanoph-2022-0600

**Published:** 2022-11-14

**Authors:** Pengning Chao, Rodrick Kuate Defo, Sean Molesky, Alejandro Rodriguez

**Affiliations:** Department of Electrical and Computer Engineering, Princeton University, Princeton, NJ 08544, USA; Department of Engineering Physics, Polytechnique Montréal, Montréal, Québec H3T 1J4, Canada

**Keywords:** fundamental limits, inverse design, local density of states, purcell enhancement

## Abstract

The electromagnetic local density of states (LDOS) is crucial to many aspects of photonics engineering, from enhancing emission of photon sources to radiative heat transfer and photovoltaics. We present a framework for evaluating upper bounds on the LDOS in structured media that can handle arbitrary bandwidths and accounts for critical wave scattering effects. The bounds are solely determined by the bandwidth, material susceptibility, and device footprint, with no assumptions on geometry. We derive an analytical expression for the maximum LDOS consistent with the conservation of energy across the entire design domain, which upon benchmarking with topology-optimized structures is shown to be nearly tight for large devices. Novel scaling laws for maximum LDOS enhancement are found: the bounds saturate to a finite value with increasing susceptibility and scale as the quartic root of the bandwidth for semi-infinite structures made of lossy materials, with direct implications on material selection and design applications.

## 1 Introduction

The electromagnetic local density of states (LDOS), a measure of local response—the electric field from a dipolar current source—at a given point in space [1], plays a central role in many nanophotonic phenomena and applications, including spontaneous [2] and stimulated [3] emission, quantum information [4], surface enhanced Raman scattering [5, 6], photovoltaics [7], radiative heat transfer [8], nonlinear frequency conversion [9], scintillation [10], to name a few. Enhancing the LDOS by nanostructuring bulk media, a persistent theme in photonics design is often achieved through the creation of optical resonances [11]: a cavity or resonator supporting a single mode of quality factor *Q* and mode volume *V* enhances the LDOS in proportion to the Purcell factor *Q*/*V*. Many classic design schemes rely on maximizing *Q* (ring resonators [12]), minimizing *V* (plasmonic nanocavities [13, 14], bow-tie antennae [15]), or a combination thereof (photonic crystal cavities [16], metal–dielectric hybrid structures [17]). Going beyond the single mode picture, multi-mode and dispersion-based effects such as exceptional points [18, 19] and slow light devices [20] have also been explored. Recently, complex devices obtained by application of large-scale structural optimization appear to combine several confinement mechanisms [21, 22], providing further improvements on device performance.

Given its relevance in optics and the ever increasing structural freedom brought on by advances in nanofabrication [23], interest in assessing limits on achievable LDOS enhancement has grown over the past few decades [24]. Spectral sum rules [25–27] pin the frequency-integrated LDOS of any system but are of limited utility in practical settings involving finite and often narrow source bandwidths. Specialized results pertaining to achievable mode quality factors [28] or mode volumes in cavity settings [29] have proven useful in many applications but are not sufficiently general to account for multi-mode effects. Passivity requirements based on achievable material response were recently used to bind both single-frequency [30] and finite-bandwidth [31] LDOS. However, passivity alone cannot fully capture the wave nature of light as constrained by Maxwell’s equations, e.g., the necessity of phase matching to achieve resonance, leading to loose limits when contributions other than those coming from evanescent fields become relevant.

In this article, we generalize and lift several limitations imposed by these prior approaches to provide expressions and predictions for the largest spectral-integrated LDOS that may be achieved in a structured medium. The derived bounds incorporate wave and material constraints imposed by Maxwell’s equations over any desired length-scale and are geometry agnostic: besides specifying the material susceptibility *χ*, a frequency window of interest, and a bounding domain that the structured medium must reside within, no further assumptions are made on device topology. We consider sources both enclosed within and external to devices, obtaining bounds that generally come within an order of magnitude of complicated structures discovered through inverse methods [32]. Furthermore, we find that the mere requirement of energy conservation is sufficient to place tight limits on large devices; we exploit this fact to derive an analytical upper bound (13) along with integral expressions and asymptotic analysis for the particular scenario of a source above a semi-infinite, structured slab. The bounds show saturation to a finite value as *χ* → ∞ and varying power-law scalings with respect to source bandwidth Δ*ω*, with the maximum LDOS transitioning from a scaling ∝Δ*ω*^−1^ to one ∝Δ*ω*^−1/4^ as material absorption begins to limit the net enhancement that any one mode may contribute.

## 2 Problem formulation

Working in dimensionless units of *ϵ*_0_ = *μ*_0_ = 1, and considering only nonmagnetic materials, the partial LDOS at frequency *ω* and position **x**′ along the direction $\hat{e}$ can be shown to be, by Poynting’s theorem [33], directly proportional to the average power *ρ*(*ω*) emitted by a harmonic dipole source $J\left(r\right){\text{e}}^{-\text{i}\omega t}={\text{e}}^{-\text{i}\omega t}\delta \left(x-x^{\prime }\right)\hat{e}$ at the same location,

(1) $$\rho \left(\omega \right)\equiv -\frac{1}{2}Re\left\{\int J^{*}\left(r\right)\cdot E\left(r\right)\;dr\right\}$$

where the electric field generated by the **J** solves Maxwell’s equations,

(2) $$\nabla \times \nabla \times E\left(r\right)-\omega^{2}\epsilon \left(r\right)E\left(r\right)=i\omega J\left(r\right).$$

Since real sources emit light over a finite bandwidth (e.g., the Planck spectrum of heated bodies or fluorescence/spontaneous emission linewidth of atoms [34]), we instead consider the more natural choice of a frequency average of *ρ*(*ω*) over a Lorentzian lineshape centered at *ω*_0_ with bandwidth Δ*ω*_src_ = *ω*_0_/2*Q*_src_ (corresponding source “quality factor” *Q*_src_):

(3a) $${\left\langle \rho \right\rangle }_{\omega_{0},Q_{\text{src}}}=\overset{\infty }{\underset{-\infty }{\int }}\frac{\Delta \omega_{\text{src}}/\pi }{{\left(\omega -\omega_{0}\right)}^{2}+\Delta \omega_{\text{src}}^{2}}\rho \left(\omega \right)\;d\omega ,$$

As pointed out in [32], such a modified figure of merit not only captures the spectral lineshape of many practical sources [34], but also offers a computational and conceptual advantage: instead of evaluating the frequency integral in (3a) directly, one can instead carry out a complex-*ω* contour integral over the upper half plane and exploit causality to convert, using the residue theorem, the spectral average ⟨*ρ*⟩ to the single complex-frequency $\rho \left(\tilde{\omega }\right)$ and an electrostatic (zero-frequency) contribution:

(4) $${\left\langle \rho \right\rangle }_{\omega_{0},Q_{\text{src}}}=\rho \left(\tilde{\omega }\equiv \omega_{0}+\frac{\omega_{0}}{2Q_{\text{src}}}i\right)+\frac{4Q_{\text{src}}}{\pi \left(4Q_{\text{src}}^{2}+1\right)\omega_{0}}\alpha .$$

Here $\alpha =\frac{1}{2}Re\left\{p_{0}\cdot E_{0}\right\}$ , where **p**_0_ is a unit amplitude electrostatic dipole and **E**_0_ the field it generates; this electrostatic contribution leads to an all-frequency integrated sum rule in the wide bandwidth limit [27, 31]. We note that (4) was previously derived in [31]; in this paper we will be focusing on the practically important case of moderate or narrow source bandwidth with *Q*_src_ ≫ 1 and thus will neglect the electrostatic term in (4) going forward.

Thus, the structural design problem for maximizing bandwidth-averaged LDOS can be formulated as

(5a) $$\underset{\epsilon \left(r;\tilde{\omega }\right)}{\max}\;\rho \left(\tilde{\omega }\right)=-\frac{1}{2}Re\left\{\int J^{*}\left(r;\tilde{\omega }\right)\cdot E\left(r;\tilde{\omega }\right)\;dr\right\}$$

given the constraints

(5b) $$\nabla \times \nabla \times E\left(r;\tilde{\omega }\right)-{\tilde{\omega }}^{2}\epsilon \left(r;\tilde{\omega }\right)E\left(r;\tilde{\omega }\right)=i\tilde{\omega }J\left(r;\tilde{\omega }\right)$$

(5c) $$\epsilon \left(r;\tilde{\omega }\right)=\left\{\begin{array}{cc} 1\text{ or }1+\chi \left(\tilde{\omega }\right)\; & r\in V\\ 1\; & r\notin V \end{array}\right.$$

where *V* is a pre-specified design region that the structure resides within and $\chi \left(\tilde{\omega }\right)$ the Fourier transform of the bulk material susceptibility evaluated at complex frequency $\tilde{\omega }$ . In later expressions we may suppress the $\tilde{\omega }$ dependence of time-harmonic variables for notation simplicity.

Due to the high dimensionality of the structural degrees of freedom *ϵ*(**r**) and the non-convex dependence of the field **E**(**r**) on *ϵ*(**r**), it is generally not possible to solve for the global optimum of (5c) [35]. However, it is possible to exploit an alternative parametrization of the problem in which the polarization density **P**(**r**) = (*ϵ*(**r**) − 1) **E**(**r**) rather than *ϵ*(**r**) serve as optimizations degrees of freedom, to obtain bounds on $\rho \left(\tilde{\omega }\right)$ applicable to *any possible structure*, given only the choices of design region *V* and material susceptibility *χ* [24]. In this context, it becomes convenient to decompose the total field into the vacuum field emitted by the source and scattered field emitted by the polarization currents:

(6) $$\begin{array}{ll} & E\left(r\right)=E_{\text{vac}}\left(r\right)+E_{\text{sca}}\left(r\right)\\ & =\frac{i}{\tilde{\omega }}\int G\left(r,r^{\prime }\right)\cdot J\left(r^{\prime }\right)\;dr^{\prime }+\int G\left(r,r^{\prime }\right)\cdot P\left(r^{\prime }\right)\;dr^{\prime } \end{array}$$

with $G\left(r,r^{\prime }\right)$ denoting the vacuum dyadic Green’s function, the solution to

(7) $$\nabla \times \nabla \times G-{\tilde{\omega }}^{2}G={\tilde{\omega }}^{2}I.$$

Note that our definition of the Green’s function has an extra global factor of ${\tilde{\omega }}^{2}$ compared to the standard definition. Similarly, the net power extracted from the dipole source can be decomposed into constant (structure-independent) vacuum and scattered-field contributions:

(8a) $$\rho =\rho_{\text{vac}}+\rho_{\text{sca}}\left(P\right),$$

(8b) $$\;\rho_{\text{vac}}=-\frac{1}{2}Re\left\{\frac{i}{\tilde{\omega }}\iint J^{*}\left(r\right)\cdot G\left(r,r^{\prime }\right)\cdot J\left(r^{\prime }\right)\;dr^{\prime }\;dr\right\},$$

(8c) $$\begin{array}{ll} \;\rho_{\text{sca}}\left(P\right) & =-\frac{1}{2}Re\left\{\iint J^{*}\left(r\right)\cdot G\left(r,r^{\prime }\right)\cdot P\left(r^{\prime }\right)\;dr^{\prime }\;dr\right\}\\ & \;=\frac{1}{2}Im\left\{\tilde{\omega }\int E_{\text{vac}}\left(r^{\prime }\right)\cdot P\left(r^{\prime }\right)\;dr^{\prime }\right\}, \end{array}$$

where in the second line of (8c) we made use of the fact that J* = **J** (the global phase of dipole source is irrelevant) and the reciprocity relation $G\left(r,r^{\prime }\right)=G^{T}\left(r^{\prime },r\right)$ . They key to formulating a shape-independent bound on *ρ* is to forgo the need for structural information by relaxing the requirement that **P** satisfy Maxwell’s equations everywhere, imposing instead a smaller subset of wave constraints [24]. Such constraints can be indeed be derived from Maxwell’s equations through a complex frequency generalization of the time-harmonic Poynting’s theorem (see [24] and SI), giving

(9) $$\begin{array}{ll} & \underset{V_{k}}{\int }E_{\text{vac}}\left(r\right)\cdot P\left(r\right)\;dr=\underset{V}{\int }P^{*}\left(r\right)\cdot \underset{V_{k}}{\int }U\left(r,r^{\prime }\right)\cdot P\left(r^{\prime }\right)\;dr^{\prime }\;dr,\\ & U\equiv \chi^{*-1}\delta \left(r-r^{\prime }\right)-G^{*}\left(r,r^{\prime }\right) \end{array}$$

where *V*_*k*_ ⊆ *V* is any spatial region within the design domain *V* and we have defined the composite operator U . Notably, (9) can be interpreted as a statement of the conservation of energy over each spatial region, requiring only specification of the allotted design footprint *V* and available susceptibility *χ*. Note also that geometric information is contained only implicitly in **P**, with material properties specified by the known complex scalar *χ*. The resulting optimization problem for the LDOS can be written as

(10a) $$\max_{P\left(r;\tilde{\omega }\right)}\;\rho_{\text{sca}}=-\frac{1}{2}Im\left\{\tilde{\omega }\left\langle E_{\text{vac}}^{*}|P\right\rangle \right\}$$

such that *∀V*_*k*_ ⊆ *V*

(10b) $$Re\text{,}Im\left\{\left\langle E_{\text{vac}}\right|I_{V_{k}}\left|P\right\rangle -\left\langle P\right|UI_{V_{k}}\left|P\right\rangle \right\}=0$$

where $I_{V_{k}}$ is the projection operator into the region *V*_*k*_. Above, we employed Dirac bra-ket notation for brevity, with $\left|a\right\rangle$ denoting the vector field **a**(**r**) over the design domain *V*, ⟨**a**|**b**⟩ = *∫*_*V*_a*(**r**) ⋅ **b**(**r**) d**r** is the conjugated inner product, and operator action $A\left|a\right\rangle =\int_{V}A\left(r,r^{\prime }\right)\cdot a\left(r^{\prime }\right)\;dr^{\prime }$ . Equation (10a) is a quadratically constrained linear program for **P**. While direct optimization is still not guaranteed to find a global optimum due to the non-convexity of certain constraints in (10b), a bound on the problem can be computed efficiently via the Lagrange dual function [24, 36, 37], hereby referred to as the dual bound. Compared to prior bounds on *ρ*_sca_ based solely on passivity [31], the constraints in (10b) contain all relevant wave physics as encoded in the EM Green’s function G ; as will be seen in later sections this leads to qualitatively tighter limits.

In principle, the maximum LDOS achievable in a structured medium is known to grow indefinitively in the single-frequency limit of Δ*ω*_src_ → 0. For instance, in lossless media, the whispering gallery modes of ring resonators and defect modes of photonic crystals exhibit exponential scaling in *Q* with increasing system size [16, 38], while their mode volumes either increase polynomially or remain constant, respectively, leading to exponential growth in the Purcell factor. Geometries with sharp tips can also give rise to field singularities and hence vanishing mode volumes [39]. However, in practice, finite source bandwidths, device footprint, and material losses limit the utility of diverging lifetimes, while fabrication imperfections and atomic-scale effects preclude realization of arbitrarily small features. As will be seen below, the imposition of finite Δ*ω*_src_ and a minimum vacuum gap *d* between source and medium regularizes such divergences, paving the way for investigations of LDOS growth characteristics in realistic settings. We also note that for finite Δ*ω*_src_ the vacuum LDOS contribution *ρ*_vac_ diverges due to contributions from high frequencies. This is an artifact of the Lorentzian lineshape chosen and has little bearing on practical applications; the results presented will thus focus on the structural contribution *ρ*_sca_ which does remain finite.

To make explicit the scale invariance of Maxwell’s equations, all lengths are given in units of the center wavelength *λ*_0_ = 2*πc*/*ω*_0_ and *ρ*_sca_ normalized by the single-frequency vacuum dipole radiation *ρ*_0_ ≡ *ρ*_vac_(*ω*_0_).

## 3 Numerical results

The dual bound can be obtained numerically for arbitrary domains via a suitable representation of the Green’s function and Maxwell operator. We do so for two standard settings: an external–dipole configuration where the dipole is adjacent to the structure, relevant for instance to gratings and solid-state defect couplers [40, 41]; and an enclosed–dipole configuration in which the dipole is surrounded by the structure, relevant to photonic crystal cavities [16], bullseye gratings [42], and bowtie antennas [15]. For computational convenience, all calculations are performed in 2*d* for both the out-of-plane (scalar) TM and in-plane (vectorial) TE electric-field polarizations. The finite-difference frequency-domain method is used to represent all relevant fields and operators (SI). Progressively tighter bounds were obtained by gradually increasing the number of constraint sub-regions *V*_*k*_ down to the computational pixel level. Inverse designs were also obtained to compare against the bounds, following the topology optimization (TO) approach detailed in [32].

**Figure 1:**
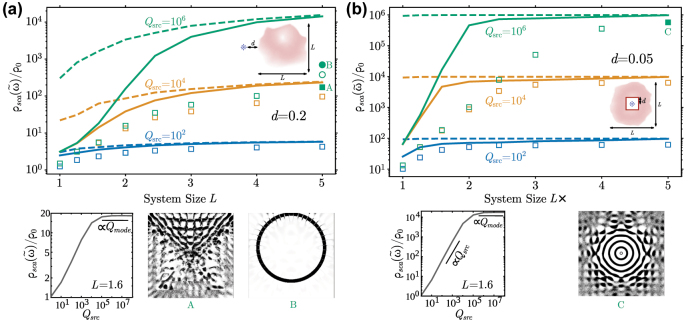
Bounds and inverse designs with TM polarization, *χ* = 4, *Q*_src_ ∈ {10^2^, 10^4^, 10^6^}, and finite design domain size *L* for (a) dipole on the side of a square design region and (b) dipole in the center of a square design region. Solid lines are bounds that are converged with respect to increasing the number of constraint subregions *V*_*k*_. Dotted lines are bounds with just the global constraint *V*_*k*_ = *V*. Squares are topology optimized structures from random initializations. The hollow circle is a ring resonator with inner and outer radii approximately 1.85 and 2.05, respectively, with the exact resonant radius requiring accuracy up to 8 significant figures to achieve the plotted enhancement (SI); the filled circle is a topology optimized structure starting from that ring resonator. LDOS enhancement as a function of *Q*_src_ for fixed *L* = 1.6 is shown beneath the main figures, along with designs corresponding to filled shapes.

Results pertaining to maximum LDOS for both external and enclosed configurations are shown in Figure 1 as a function of the design footprint. Since the vacuum LDOS diverges when integrated against a Lorentzian lineshape [25, 31, 32], this and subsequent results pertain only to the structural contribution *ρ*_sca_, defining the enhancement factor in relation to the single-frequency vacuum emission *ρ*_0_ ≡ *ρ*_vac_(*ω*_0_). In both settings the medium is assumed to be lossless, Im *χ* = 0. For a fixed *Q*_src_, the bounds are seen to grow exponentially before saturating with increasing domain size *L*. Conversely, for fixed *L* the bounds grow linearly before saturating with increasing *Q*_src_. Both observations are consistent with a resonant enhancement mechanism: since the finite vacuum gap *d* precludes field divergences in the vicinity of the dipole, thereby imposing an upper bound on its coupling to any mode, one would expect improvements in the Purcell factor to be mainly driven by growth in the modal lifetimes. Supposing a system with response dominated by a single mode of quality factor *Q*_mode_ = *ω*_0_/2Δ*ω*_mode_, (3a) yields a frequency-integrated LDOS that scales as

(11) $$\overset{\infty }{\underset{-\infty }{\int }}\frac{\Delta \omega_{\text{src}}/\pi }{\omega^{2}+\Delta \omega_{\text{src}}^{2}}\frac{\Delta \omega_{\text{mode}}/\pi }{\omega^{2}+\Delta \omega_{\text{mode}}^{2}}\;d\omega =\frac{Q_{\text{src}}Q_{\text{mode}}}{2\pi \omega_{0}\left(Q_{\text{src}}+Q_{\text{mode}}\right)}.$$

Thus, this modal picture predicts linear growth ∝*Q*_src_ in (3a) so long as the system supports a sufficiently long-lived mode, *Q*_mode_ ≫ *Q*_src_, eventually saturating to a value ∝*Q*_mode_ whenever *Q*_mode_ ≪ *Q*_src_. In the absence of material dissipation, the highest achievable *Q*_mode_ is constrained solely by radiative losses and, as confirmed by our bound calculations, known to scale at least exponentially with *L* in ring-resonator and photonic-crystal cavities. Comparing our bounds (solid lines) against inverse designs (squares), one observes remarkable alignment, with bounds and designs often coming within an order of magnitude of each other. The biggest performance gaps occur at high *Q*_src_ in the external configuration and can at least be partially attributed to TO getting trapped in local optima that, regardless of exhaustive initial conditions, underperform simple ring resonator geometries. Even starting with ring resonators as initial seeds, TO was only able to make modest improvements. In contrast, Bragg–onion type structures discovered by TO in the cavity setting are seen to tightly approach corresponding bounds. Comparing the high degree of precision needed to specify the dimensions of the ring resonator against the relative robustness of photonic bandgap confinement [43], we hypothesize that attaining high-performing designs for the external configuration at narrow bandwidths requires resonances based on sensitive interference cancellation. This leads to an ill-behaved optimization landscape with many subpar local optima to get stuck in, with ring resonators as an example of a class of high-performing designs not easily discoverable via brute-force optimization.

Finally, the observed dependence of the dual bound on the number and size of subregion constraints (10b) reveals two important features: first, the necessity of imposing many subwavelength constraint regions (SI) for the bounds to exhibit saturation as *Q*_src_ → ∞; second, the fact that a single, global energy-conservation constraint appears sufficient to produce tight bounds when either device sizes or source bandwidths become sufficiently large. This suggests that subregion constraints are necessary to adequately “resolve” wave effects, including phase-matching restrictions, which limit mode confinement in finite systems; conversely, when system sizes no longer limit the highest achievable *Q*_mode_ in relation to *Q*_src_, the latter and not the former becomes a bottleneck for further enhancements.

## 4 Large-size semi-analytics

We now exploit the remarkable success of global energy conservation in describing large system behavior to obtain a semi-analytical bound on the maximum LDOS above a semi-infinite structure, i.e., the *L* → ∞ limit of the external–dipole configuration depicted in Figure 1a. As shown in SI, the solution of the dual problem under both resistive and reactive energy conservation constraints (10b) can be mapped to a family of solutions involving a unitary phasor parametrized by the single constraint angle *θ*, leading to the modified problem:

(12a) $$\max_{P\left(r;\tilde{\omega }\right)}\;\rho_{\text{sca}}=-\frac{1}{2}Im\left\{\tilde{\omega }\left\langle E_{\text{vac}}^{*}|P\right\rangle \right\}$$

such that for **P** ∈ *V*,

(12b) $$Im\left\{{\text{e}}^{\text{i}\theta }\left\langle E_{\text{vac}}|P\right\rangle \right\}-\left\langle P\right|\text{Asym}\left({\text{e}}^{\text{i}\theta }U\right)\left.\left|P\right\rangle \right)=0$$

where $\text{Asym}\left(A\right)=\left(A-A^{†}\right)/2i$ is the Hermitian anti-symmetric component of the operator A . Owing to its simplicity, the solution to this dual bound for any given *θ* can be written explicitly as

(13) $$\begin{array}{l} \rho_{\text{sca}}\leq \frac{1}{4}Re\left\{{\left(-{\tilde{\omega }}^{*}{\text{e}}^{\text{i}\theta }\left|E_{\text{vac}}^{*}\right\rangle +|\tilde{\omega }|\left|E_{\text{vac}}\right\rangle \right)}^{†}\right.\\ \left.\text{Asym}{\left({\text{e}}^{\text{i}\theta }U\right)}^{-1}\left|E_{\text{vac}}\right\rangle \right\}, \end{array}$$

which notably, depends only on the analytically known quantities **E**_vac_, *χ*, and U . The tightest dual bound consistent with (13) can thus be obtained numerically by carrying out an additional optimization over *θ*, restricted so that $\text{Asym}\left({\text{e}}^{\text{i}\theta }U\right)$ is positive definite (SI). Further analytical insight can be gleaned by expanding the operators and fields in a spectral basis conforming to the symmetry of the design domain: for a half-space enclosure, the natural choice is a Fourier basis $e^{ik_{\|}x_{\|}}$ parametrized by the wavevector *k*_‖_ parallel to the half-space surface. Carrying out this expansion for the incident field $\left|E_{\text{vac}}\right\rangle$ yields,

(14) $$\left|E_{\text{vac}}\right\rangle =-\frac{\tilde{\omega }}{2\sqrt{2\pi }}\overset{\infty }{\underset{-\infty }{\int }}\frac{e^{ik_{\|}x}}{\sqrt{2\pi }}\frac{1}{k_{⊥}}e^{ik_{⊥}x_{⊥}}e^{ik_{⊥}d}dk_{\|}$$

where $k_{⊥}=\sqrt{{\tilde{\omega }}^{2}-k_{\|}^{2}}$ and Im(*k*_⊥_) ≥ 0. For each conserved *k*_‖_, the inverse operator image $\text{Asym}{\left({\text{e}}^{\text{i}\theta }U\right)}_{k_{\|}}^{-1}\cdot e^{ik_{⊥}x_{⊥}}$ can be similarly expanded and evaluated explicitly as the sum of two complex sinusoids, leading to

(15)  ρsca≤minθ116π∫0∞Re−ω~3eiθe2ik⊥dk⊥2R1r1−ik⊥+R2r2−ik⊥+|ω~|3e−2Im(k⊥)d|k⊥|2R1r1+ik⊥*+R2r2+ik⊥*dk‖

where the closed-form complex coefficients *R*_1,2_(*k*_‖_; *θ*) and decay constants *r*_1,2_(*k*_‖_; *θ*) are given in the SI.

The main challenge in evaluating (13) comes from the need to compute the inverse image of $\text{Asym}\left({\text{e}}^{\text{i}\theta }U\right)={\left[Im\left\{{\text{e}}^{\text{i}\theta }/\chi^{*}\right\}+\left.\text{Asym}\left({\text{e}}^{-\text{i}\theta }G\right)\right)\right]}^{-1}$ , which contains constraints on wave propagation via its dependence on the vacuum Green’s function G . In that regard, it is useful to consider an expansion of (13) in orders of $\text{Asym}\left({\text{e}}^{-\text{i}\theta }G\right)$ (after using the Cauchy–Schwartz inequality to relax the term involving $E_{\text{vac}}^{*}$ (SI)):

(16) $$\begin{array}{ll} & \rho_{sca}\leq \frac{\left|\tilde{\omega }\right|}{2}\left\langle E_{\text{vac}}\right|{\left[Im\left\{\frac{{\text{e}}^{\text{i}\theta }}{\chi^{*}}\right\}+\text{Asym}\left({\text{e}}^{-\text{i}\theta }G\right)\right]}^{-1}\left|E_{\text{vac}}\right\rangle \\ & =\frac{\left|\tilde{\omega }\right|}{2}\left\langle E_{\text{vac}}\right|Im{\left\{\frac{{\text{e}}^{\text{i}\theta }}{\chi^{*}}\right\}}^{-1}\left[I-\frac{\text{Asym}\left({\text{e}}^{-\text{i}\theta }G\right)}{Im\left\{{\text{e}}^{\text{i}\theta }/\chi^{*}\right\}}+\cdots \;\right]\left|E_{\text{vac}}\right\rangle \\ & \leq \frac{\left|\tilde{\omega }\right|}{2}Im{\left\{\frac{{\text{e}}^{\text{i}\theta }}{\chi^{*}}\right\}}^{-1}\left\langle E_{\text{vac}}|E_{\text{vac}}\right\rangle . \end{array}$$

The zeroth-order approximation given in (16) is especially simple to evaluate as it only depends on *χ*, and will hereafter be referred to as a material bound. A special yet sub-optimal value of *θ* in (16) recovers prior LDOS bounds based on passivity (SI) [31]. Notably, this zeroth order approximation becomes loose whenever $Im{\left\{{\text{e}}^{\text{i}\theta }/\chi^{*}\right\}}^{-1}\text{Asym}\left({\text{e}}^{-\text{i}\theta }G\right)$ is large as compared to I ; this is the case for large |*χ*| or large design domains (e.g., the half-space) where AsymG dominates. Intuitively, terms containing the vacuum Green’s function capture physical limitations imposed by multiple scattering, screening, and the finite speed of light, which limit achievable LDOS.

**Figure 2:**
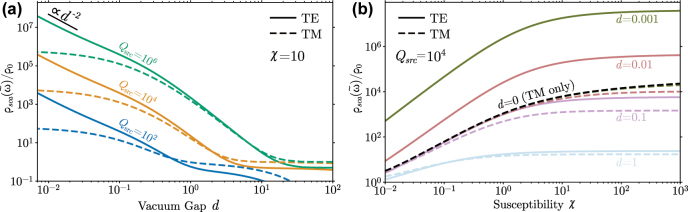
Maximum LDOS enhancement near a half-space design region as a function of (a) separation distance and (b) real material susceptibility *χ* for lossless dielectrics. While (b) plots bounds for lossless dielectrics, the material independent limit as *χ* → ∞ is a bound for general lossy *χ* as well (SI).

Figure 2 shows upper bounds on the LDOS for both TM and TE sources, obtained by evaluating (15). In the near-field limit of *d* → 0, the TM bounds are found to asymptote to a constant while the TE bounds scale ∝1/*d*^2^, in agreement with a detailed asymptotic analysis of the evanescent *k*_‖_ → ∞ behavior of the integrand in (15) (SI), and matching prior results based on passivity [31]. The constant asymptote observed for TM sources as they approach the device reflects a known artifact of 2*d* scalar electromagnetism, which precludes non-integrable field singularities at sharp corners [44]. In contrast, the 1/*d*^2^ scaling of TE sources confirms the known divergence in the energy concentration of a vector dipole in its near field, with the exponent of *d* tied to the number of spatial dimensions; note that the maximum LDOS for a 3*d* source grows ∝1/*d*^3^ as *d* → 0 (SI). While Figure 2 shows data only for lossless dielectrics, the asymptotics in the SI apply equally as well to metals with Re(*χ*) < 0, which exhibit the same scaling with *d*.

In the opposite far-field limit of *d* → ∞ and for finite *Q*_src_, the bounds ultimately tend toward zero, reflecting a kind of space–bandwidth constraint on the ability of structuring to affect far-field emission over a finite bandwidth. One can, however, observe plateaus occurring at intermediate regimes, *ωd*/*c* ≪ *Q*_src_, wherein structuring can efficiently reflect traveling planewaves back onto the dipole position. Achievable enhancements in this regime can be shown to decay $\propto e^{-d/Q_{\text{src}}}$ , manifesting as plateaus in the various plots of Figure 2b. Only in the strictly single-frequency limit *Q*_src_ → ∞ do far-field LDOS contributions tend toward constants, *ρ*_0_/2 and *ρ*_0_ for TE and TM sources, respectively, independently of separation. The importance of properly capturing relevant wave interference effects in these far-field regimes becomes evident when analyzing corresponding predictions for material bounds [31], which exhibit drastically inflated $\propto Q_{\text{src}}^{2}$ scaling in this half-space setting (SI).

Asymptotic analysis of (15) supported by Figure 2b reveal that LDOS enhancements saturate to finite values as $\left|\chi \right|\to \infty$ for both dielectrics and metals (SI), in contrast to prior bounds which grow indefinitely with increasing material response [31]. This is somewhat surprising given that larger *χ* implies a larger (potentially infinite) density of states within the material itself; ultimately, multiple scattering and screening effects lead to restrictions on the possible field localization at the source location that cannot be overcome with clever structuring. Similar conclusions have been reached concerning the efficacy of hyperbolic metamaterials [45] at enhancing the LDOS, though we emphasize that our results are more general and make no assumption on the particulars of the device structure. It is also worth noting that the saturation characteristics of the full bounds as a function of *d* are distinct for the TM and TE polarizations. For TM, reducing *d* increases the relative advantage of stronger materials; *ρ*(*χ* → ∞) ∝ log(1/*d*) as *d* → 0 whereas *ρ*(*χ* < ∞) is finite as *d* → 0 (SI). In contrast, for TE the saturation behavior for large |*χ*| scales uniformly with *d*; decreasing separation does not increase the relative advantage of stronger materials.

With regard to bandwidth scaling, the broad structural freedom afforded by an infinite design domain coupled with the lack of material dissipation allows for creation of resonances with arbitrarily small radiative loss rates, *Q*_mode_ → ∞, leading to the same ∝*Q*_src_ dependence observed in Figure 1. The situation changes in dissipative media where mode lifetimes $Q_{\text{mode}}={\left(\frac{1}{Q_{\text{abs}}}+\frac{1}{Q_{\text{rad}}}\right)}^{-1}$ contain both radiative *Q*_rad_ and absorptive *Q*_abs_ contributions, with *Q*_abs_ ∝ 1/Im(*χ*) denoting the proportion of stored energy dissipated in the medium per unit cycle. Hence, under finite absorption, *Q*_abs_ sets a bound on the highest achievable mode lifetime, with (11) suggesting a saturating LDOS ∝*Q*_abs_ as *Q*_src_ → ∞.

**Figure 3:**
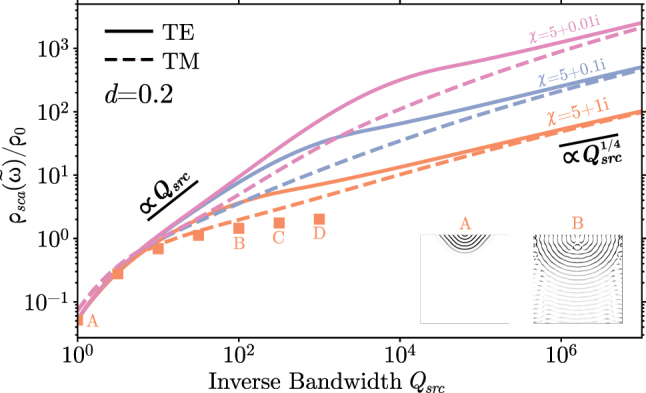
Maximum LDOS enhancement near a halfspace design region as a function of the source bandwidth for dielectric Re(*χ*) = 5. Squares are the performance of TM inverse designs for *χ* = 5 + 1*i*, with points between *A* and *B* (inclusive) over a 10 × 10 design region and *C*, *D* over a 3 × 200 design region. Insets *A* and *B* show the structures found via TO for *Q*_src_ = 1 and *Q*_src_ = 100, respectively. Additional data pertaining to metallic *χ* is included in the SI.

In contrast, Figure 3 shows that the bounds exhibit a transition from the linear ∝*Q*_src_ scaling of a wide-bandwidth source coupled to a single resonance toward *diverging* response $\propto Q_{\text{src}}^{1/4}$ as *Q*_src_ → ∞, with the transition taking place as *Q*_src_ → *Q*_abs_. Intuitively, in this regime wherein the lifetime of a single resonance is capped, one might expect the optimal design strategy to shift toward exploiting degeneracies. One class of geometry capable of achieving high modal degeneracies are waveguides, e.g., coupled cavities or photonic crystal gratings, which at least in 1*d* are known to generate van Hove singularities in the density of states [46]. Generally, a spectral singularity of the form |*ω* − *ω*_0_|^−*α*^, 0 < *α* < 1 will yield a Lorentzian-averaged LDOS that scales as

(17) $$\overset{\infty }{\underset{-\infty }{\int }}\frac{\Delta \omega_{\text{src}}/\pi }{{\left(\omega -\omega_{0}\right)}^{2}+\Delta \omega_{\text{src}}^{2}}\frac{1}{|\omega -\omega_{0}{|}^{\alpha }}\;d\omega \propto \frac{1}{{\left(\Delta \omega_{\text{src}}\right)}^{\alpha }}\propto Q_{\text{src}}^{\alpha }.$$

The bounds for the 2*d* half-space configuration thus suggest the possibility of near-field absorbers supporting quartic “band-edge” dispersions capable of efficiently extracting evanescent fields, resulting in singularities of the form |*ω* − *ω*_0_|^−1/4^ (anomalous quartic ”slow” group-velocity dispersions have been studied, for instance, in the context of waveguide solitons [47]). Indeed, TO for *χ* = 5 + 1*i* discovers structures resembling adiabatic gratings (Figure 3 insets) with performances that approach the bounds and continue growing with increasing *Q*_src_ beyond what is expected of single mode enhancement given *Q*_abs_ on the order of 10. The remaining gap between inverse design and bound for narrow bandwidths is partially due to the finite size of the TO design domain, with finite domain bounds saturating as *Q*_src_ → ∞ (SI); further investigation is needed to clarify the tightness of the bound and the feasibility of engineering semi-infinite structures with the requisite quartic root spectral singularity.

## 5 Summary

We proposed an improved framework for evaluating upper bounds on the LDOS in structured media, and study in detail the effects of source bandwidth, material susceptibility, and device footprint. The bounds provide a strong top–down complement to bottom–up design in at least two ways: First, they are useful in quantifying the optimality of existing “best” structures while diagnosing potential areas of improvement: as seen in narrow bandwidth regimes, existing formulations of inverse design fail to converge upon structures with performance close to the global optimum, often outperformed by traditional ring resonators. Second, they allow studies of fundamental scaling characteristics without prior assumptions on device topology.

For finite bandwidths, the quick saturation of the bounds with design size indicate that a device footprint of a few wavelengths is generally enough to achieve near-optimal performance. The observed saturation with increasing susceptibility has direct implications for material choice: a weak material response can be mitigated given sufficient design size and judicious structuring; conversely there are diminishing returns associated with seeking large absolute values of the susceptibility, raising the importance of other concerns such as material loss and dispersion characteristics. The impact of material loss was also evaluated in the context of maximum LDOS above a semi-infinite structure, showing a transition from the intuitive linear ∝*Q*_src_ scaling expected of single-mode resonant enhancement to a less obvious $\propto Q_{\text{src}}^{1/4}$ dependence given absorptive materials, inviting further studies into the associated dispersion engineering mechanisms making such scaling possible.

While only results for dielectrics (Re(*χ*) > 0) where shown in the figures, the framework can handle metals (Re(*χ*) < 0) just as well. Metals may have an edge over dielectrics for electrically small design domains due to material resonances allowing for highly subwavelength confinement at the cost of high loss [48]. For large design domains such as the half-space, the bounds for metals have the same scaling with regards to source bandwidth and separation, and the existence of a material-independent limit blunts the advantage of the large $\left|\chi \right|$ of metals relative to their drawback of high loss.

Code for the computation of halfspace bounds makes use of the Python arbitrary precision arithmetic package mpmath [49] and is given at [50].

## Supplementary Material

Supplementary Material Details
