# Supplementary material for: Maximum electromagnetic local density of states via material structuring
Source: Nanophotonics. 2022 Nov 14;12(3):549–57. doi: 10.1515/nanoph-2022-0600 (PMC11501631; doi:10.1515/nanoph-2022-0600)
Supplement: Supplementary file 1 — Supplementary Material Details [file j_nanoph-2022-0600_suppl.pdf]

Maximum Electromagnetic Local Density of States via Material Structuring: Supplementary Information

Pengning Chao*, Rodrick Kuate Defo*, Sean Molesky, Alejandro Rodriguez

November 2, 2022

Contents

1 Computational Details **3**

2 Dependence of bounds on number of subregion constraints **3**

3 Ring resonator LDOS enhancement **4**

4 Bandwidth scaling of half-space LDOS bounds for metals **5**

5 Bandwidth saturation of finite size LDOS bounds **5**

6 Differences in notation in supplementary information compared to the main text **6**

7 Derivation of power conservation constraints via complex Poynting's theorem **6**
7.1 Complex Poynting's theorem for time harmonic fields and complex ω 6
7.2 Generalized energy conservation constraints 7

8 Lagrangian duality given global constraints **9**

9 Spectral analysis of Green's function **10**

10 Spectral Analysis of Asym($p\mathbb{U}$) **11**
10.1 Lossless dielectrics 12

11 Global constraint bounds for 2D TM dipole near a half-space **13**
11.1 TM Green's function and dipole field 13
11.2 Simplification of (S24) 13
11.3 Evaluation of $\text{Asym}(p\mathbb{U}^{TM})^{-1}\left|\mathbf{E}_v^{TM}\right\rangle$ 14
11.4 TE Green's function and dipole field 17

12 Asymptotic analysis **18**
12.1 TM bandwidth scaling 18
12.1.1 Traveling wave contribution 18
12.1.2 Evanescent wave contribution 20

12.2 TM material Scaling 21
12.3 TM separation scaling 21
12.3.1 Finite χ 22
12.3.2 Material independent bounds 22
12.4 TE separation scaling 22
12.4.1 3D separation scaling 23

13 Comparison to passivity bounds **23**
13.1 Bandwidth scaling of the passivity bounds 24

1 Computational Details

Inverse design in this work was performed using the ceviche Maxwell FDFD solver [1] combined with the method of moving asymptotes algorithm as implemented in the non-linear optimization package NLOPT [2].

Finite size LDOS bounds were computed using an in-house FDFD code to represent the Green's function and the electric fields over the design domain. The dual function is optimized with an in-house implementation of BFGS; for details on the computation of the dual gradient see [3].

2 Dependence of bounds on number of subregion constraints

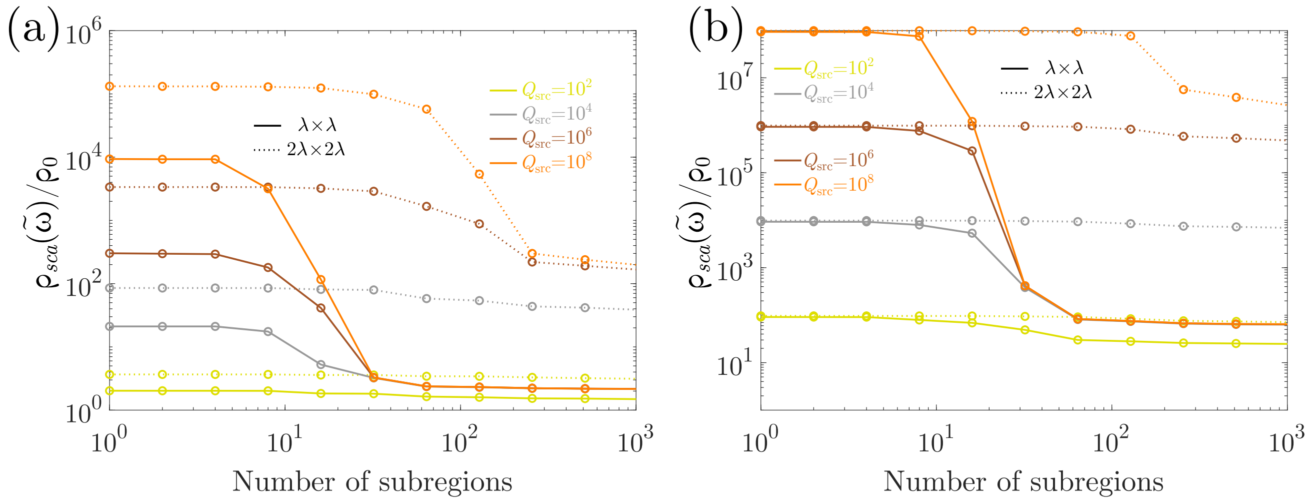

Figure S1: Plot showing the computed LDOS dual bounds as a function of the number of subregion constraints used. The subplots (a) and (b) correspond to the exterior dipole and interior dipole geometries shown in Fig. 1 of the main text. Solid lines are for system size $L = 1$ and dotted lines are for system size $L = 2$.

Fig. S1 shows how the bounds depend on the number of subregion constraints used. Generally there is a rapid transition of many orders of magnitude when the number of subregions reaches a critical value before convergence of the bounds. For larger Q_{src} the transition is steeper, indicating the importance of subregion constraints for capturing the effects of radiative loss. For larger system size L, the transition is shallower: as stated in the main text, in the limit of large L global constraints alone are sufficient to achieve tight bounds.

3 Ring resonator LDOS enhancement

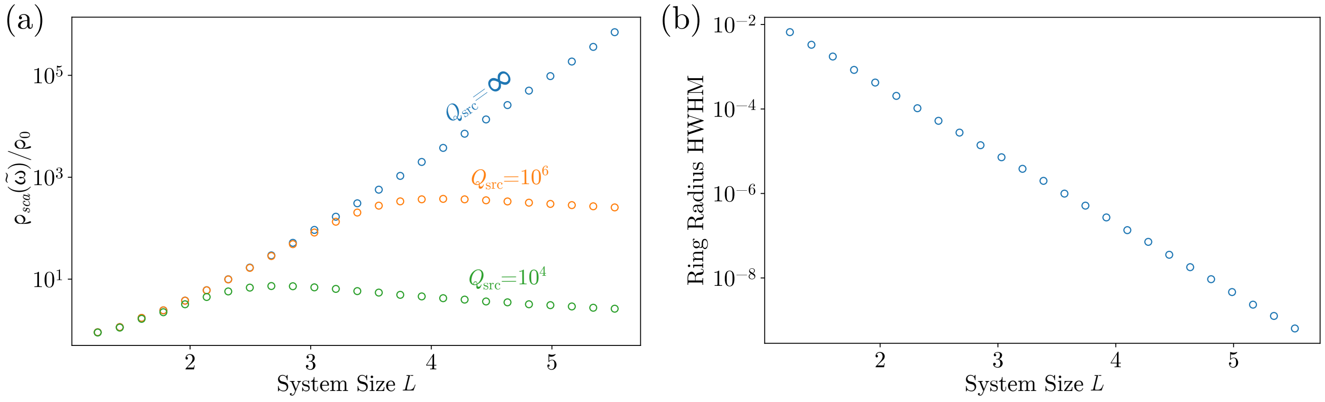

Figure S2: (a) LDOS enhancement of ring resonators with a fixed ring width of 0.2 and TM dipole source separated 0.2 away from the ring. The system size L is 2 times the outer radius of the rings. (b) At $Q_{src} \to \infty$, the change in the ring radius Δr such that the LDOS enhancement drops by a half.

In the main text we presented a specific ring resonator design with a ring width of 0.2 that outperformed topology optimization from multiple random initializations. Here are extra data on the performance of ring resonators as a function of size for various Q_{src}. In the single frequency limit, we see the exponential scaling of LDOS enhancement as a function of ring size; for finite Q_{src} there is a point where larger rings have higher Q_{mode} but do not lead to larger bandwidth-averaged LDOS. For the $Q_{src} = 10^6$ design shown in the main text the outer ring diameter is approximately 4.1; Fig. S2b indicates that we require around 8 significant figures of accuracy on the ring size to fall within 50% of the maximum performance.

4 Bandwidth scaling of half-space LDOS bounds for metals

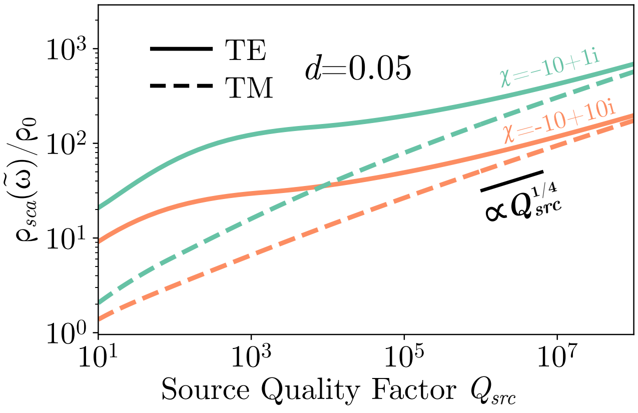

Figure S3: Half-space LDOS bounds as a function of Q_{src} for metallic material response $\chi = -10 + 1i$ and $\chi = -10 + 10i$ and source-design separation $d = 0.05$.

For metallic material response $\text{Re}(\chi) < 0$, the half-space bounds also exhibit a $Q_{src}^{1/4}$ scaling in the narrow bandwidth limit $Q_{src} \to \infty$. Per [7], the requirement of passivity implies that the complex frequency $\chi(\tilde{\omega})$ satisfies $\text{Im}(\tilde{\omega}\chi) > 0$, which truncates the wide bandwidth (small Q_{src}) portion of Fig. S3.

5 Bandwidth saturation of finite size LDOS bounds

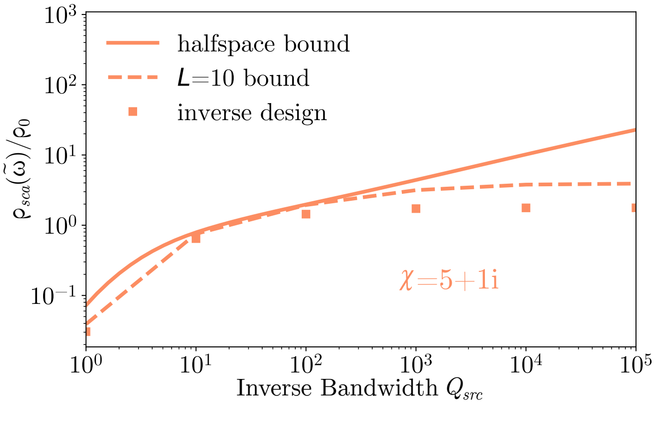

Figure S4: LDOS bounds as a function of Q_{src} for $\chi = 5 + 1i$.

In Fig. 3 of the main text, the inverse designs are performed over a finite design domain of size 10 by 10, and the LDOS enhancement saturates with increasing Q_{src}. Fig. S4 shows that this saturation is also present we you compute the bounds for the finite design domain; continued $Q_{src}^{1/4}$ scaling is seen just for the semi-infinite halfspace bounds.

6 Differences in notation in supplementary information compared to the main text

In the following sections, some quantities and variables use a different notation than what is written in the main text. This is predominantly for conciseness in writing down long derivations. For convenience a complete list of the differences in notation is listed here; each individual notation deviation will also be noted at its first occurrence in the SI.

- The vacuum field $\mathbf{E}_{vac}$ in main text is referred to as $\mathbf{E}_v$ in the SI.
- Use of k_x and k_y instead of $k_\parallel$ and $k_\perp$ for half-space bounds, with the $\hat{x}$ direction set as parallel to the half-space surface and $\hat{y}$ direction perpendicular to it.
- the complex parameters $R_{1,2}$ and $r_{1,2}$ in the main text are replaced with $R_\pm$ and $r_\pm$ in the SI, where the $\pm$ subscripts have more background context within the derivation.
- With dimensionless units $c = 1$, $\tilde{k} = \tilde{\omega}/c$ will often be used in place $\tilde{\omega}$ in the context of spatial Fourier integrals.

7 Derivation of power conservation constraints via complex Poynting's theorem

Here we present a derivation of the complex scattering constraints used via the complex Poynting's Theorem. For alternative derivations see early work [...].

7.1 Complex Poynting's theorem for time harmonic fields and complex ω

In prior literature the complex Poynting's Theorem for time harmonic fields is presented given a real angular frequency ω. The generalization to a complex ω is straightforward and written explicitly here for clarity. All fields have (complex) harmonic time dependence $e^{-i\omega t}$, with the harmonic time Maxwell's equations

$$\nabla \cdot \mathbf{D} = \rho_f \tag{S1a}$$
$$\nabla \cdot \mathbf{B} = 0 \tag{S1b}$$
$$\nabla \times \mathbf{E} = i\omega \mathbf{B} \tag{S1c}$$
$$\nabla \times \mathbf{H} = \mathbf{J}_f - i\omega \mathbf{D} \tag{S1d}$$

and linear constitutive relations $\mathbf{D}(\mathbf{r}) = \epsilon_0 \overline{\epsilon}_r(\mathbf{r})\mathbf{E}(\mathbf{r})$ and $\mathbf{B}(\mathbf{r}) = \mu_0 \overline{\mu}_r(\mathbf{r})\mathbf{H}(\mathbf{r})$, with $\overline{\epsilon}(\mathbf{r})$ and $\overline{\mu}(\mathbf{r})$ being tensor fields to allow for anisotropy.
Define the complex Poynting vector

$$\mathbf{S} = \mathbf{E} \times \mathbf{H}^* \tag{S2}$$

Taking its divergence yields

$$\nabla \cdot \mathbf{E} \times \mathbf{H}^* = \mathbf{H}^* \cdot \nabla \times \mathbf{E} - \mathbf{E} \cdot \nabla \times \mathbf{H}^* \tag{S3}$$

Now applying Maxwell's equations gives the differential form of the complex Poynting's theorem

$$\begin{aligned}\boldsymbol{\nabla}\cdot\mathbf{E}\times\mathbf{H}^* &= i\omega\mathbf{H}^*\times\mathbf{B} - i\omega^*\mathbf{E}\cdot\mathbf{D}^* - \mathbf{E}\cdot\mathbf{J}^* \\ &= i\omega\mathbf{H}^*\cdot\overline{\mu}\cdot\mathbf{H} - i\omega^*\mathbf{E}\cdot\overline{\epsilon}^*\cdot\mathbf{E}^* - \mathbf{E}\cdot\mathbf{J}^*\end{aligned} \tag{S4}$$

The corresponding integral form is

$$\int_{\partial V} \mathrm{d}\sigma\cdot(\mathbf{E}\times\mathbf{H}^*) = i\omega\int_V \mathbf{H}^*\cdot\overline{\mu}\cdot\mathbf{H}\,\mathrm{d}V - i\omega^*\int_V \mathbf{E}\cdot\overline{\epsilon}^*\cdot\mathbf{E}^*\,\mathrm{d}V - \int_V \mathbf{E}\cdot\mathbf{J}^*\,\mathrm{d}V \tag{S5}$$

in the case where ω is real we have

$$\int_{\partial V} \mathrm{d}\sigma\cdot(\mathbf{E}\times\mathbf{H}^*) = i\omega\int_V (\mathbf{H}^*\cdot\overline{\mu}\cdot\mathbf{H} - \mathbf{E}\cdot\overline{\epsilon}^*\cdot\mathbf{E}^*)\,\mathrm{d}V - \int_V \mathbf{E}\cdot\mathbf{J}^*\,\mathrm{d}V \tag{S6}$$

which is a more familiar form.

7.2 Generalized energy conservation constraints

To arrive at the generalized energy conservation constraints, we start with a scattering theory framework in which an initial free current source $\mathbf{J}_v$ produces the fields $\mathbf{E}_v$, $\mathbf{H}_v$ in vacuum. These initial fields interact with a scatterer, producing polarization currents $\mathbf{J}_s$ within the scatterer that generate scattered fields $\mathbf{E}_s$, $\mathbf{H}_s$. For simplicity, we assume that the scatterer is non-magnetic, i.e., $\overline{\mu} = \mu_0$, and that the electric permittivity is local but may be anisotropic: $\overline{\epsilon}_r = \epsilon_0(\mathbb{I} + \mathbb{I}_s\overline{\chi}\mathbb{I}_s)$. The net result is a total field $\mathbf{E}_t = \mathbf{E}_v + \mathbf{E}_s$, $\mathbf{H}_t = \mathbf{H}_v + \mathbf{H}_s$. The complex Poynting theorem then applies in three settings: to the free current and initial fields $(\mathbf{J}_v, \mathbf{E}_v, \mathbf{H}_v)$ in vacuum, to the polarization current and scattered fields $(\mathbf{J}_s, \mathbf{E}_s, \mathbf{H}_s)$ in vacuum, and to the free current and total fields $(\mathbf{J}_v, \mathbf{E}_t, \mathbf{H}_t)$.

We consider as our region of interest some subset V_k of the design region. For $(\mathbf{J}_v, \mathbf{E}_v, \mathbf{H}_v)$ in vacuum we have

$$\int_{\partial V_k} \mathrm{d}\sigma\cdot(\mathbf{E}_v\times\mathbf{H}_v) = i\omega\mu_0\int_{V_k} \mathbf{H}_v^*\cdot\mathbf{H}_v\,\mathrm{d}V - i\omega^*\epsilon_0\int_{V_k} \mathbf{E}_v\cdot\mathbf{E}_v^*\,\mathrm{d}V. \tag{S7}$$

For $(\mathbf{J}_s, \mathbf{E}_s, \mathbf{H}_s)$ in vacuum we have

$$\int_{\partial V_k} \mathrm{d}\sigma\cdot(\mathbf{E}_s\times\mathbf{H}_s) = i\omega\mu_0\int_{V_k} \mathbf{H}_s^*\cdot\mathbf{H}_s\,\mathrm{d}V - i\omega^*\epsilon_0\int_{V_k} \mathbf{E}_s\cdot\mathbf{E}_s^*\,\mathrm{d}V - \int_{V_k} \mathbf{E}_s\cdot\mathbf{J_s}^*\,\mathrm{d}V. \tag{S8}$$

For $(\mathbf{J}_v, \mathbf{E}_t, \mathbf{H}_t)$ with the scatterer we have (noting that the free current is situated outside of the design region)

$$\begin{aligned}&\int_{\partial V_k} \mathrm{d}\sigma\cdot((\mathbf{E}_v+\mathbf{E}_s)\times(\mathbf{H}_v+\mathbf{H}_s)) \\ &= i\omega\mu_0\int_{V_k} (\mathbf{H}_v^*+\mathbf{H}_s^*)\cdot(\mathbf{H}_v+\mathbf{H}_s)\,\mathrm{d}V - i\omega^*\epsilon_0\int_{V_k} (\mathbf{E}_v+\mathbf{E}_s)\cdot(\mathbb{I}+\mathbb{I}_s\overline{\chi}^*\mathbb{I}_s)\cdot(\mathbf{E}_v^*+\mathbf{E}_s^*)\,\mathrm{d}V.\end{aligned} \tag{S9}$$

Subtracting (S7) and (S8) from (S9) gives

$$\begin{aligned}
&\int_{\partial V_k} \mathrm{d}\sigma \cdot (\mathbf{E}_v \times \mathbf{H}_s + \mathbf{E}_s \times \mathbf{H}_v) \\
&= i\omega\mu_0 \int_{V_k} (\mathbf{H}_v^* \cdot \mathbf{H}_s + \mathbf{H}_s^* \cdot \mathbf{H}_v)\, \mathrm{d}V \\
&\quad - i\omega^*\epsilon_0 \int_{V_k} \big[\mathbf{E}_v \cdot \mathbb{I}_s\overline{\chi}^*\mathbb{I}_s \cdot \mathbf{E}_v^* + \mathbf{E}_s \cdot \mathbb{I}_s\overline{\chi}^*\mathbb{I}_s \cdot \mathbf{E}_s^* + \mathbf{E}_v \cdot (\mathbb{I} + \mathbb{I}_s\overline{\chi}^*\mathbb{I}_s) \cdot \mathbf{E}_s^* + \mathbf{E}_s \cdot (\mathbb{I} + \mathbb{I}_s\overline{\chi}^*\mathbb{I}_s) \cdot \mathbf{E}_v^*\, \mathrm{d}V \\
&\quad + \int_{V_k} \mathbf{E}_s \cdot \mathbf{J_s}^*\, \mathrm{d}V.
\end{aligned} \tag{S10}$$

To further simplify this expression, we investigate the cross term

$$\mathbf{H}_v^* \cdot \mathbf{H}_s = \frac{1}{\mu_0^2|\omega|^2}(\boldsymbol{\nabla} \times \mathbf{E}_s) \cdot (\boldsymbol{\nabla} \times \mathbf{E}_v^*) = \frac{1}{\mu_0^2|\omega|^2}\boldsymbol{\nabla} \boldsymbol{\cdot} (\mathbf{E}_s \times \boldsymbol{\nabla} \times \mathbf{E}_v^*) + \frac{1}{\mu_0^2|\omega|^2}\mathbf{E}_s \cdot \boldsymbol{\nabla} \times \boldsymbol{\nabla} \times \mathbf{E}_v^*$$

where we have used the identity

$$(\boldsymbol{\nabla} \times \mathbf{A}) \cdot \mathbf{B} = \boldsymbol{\nabla} \boldsymbol{\cdot} (\mathbf{A} \times \mathbf{B}) + \mathbf{A} \cdot (\boldsymbol{\nabla} \times \mathbf{B}).$$

Now $\mathbf{E}_v$ satisfies the vector wave equation

$$\begin{aligned}
\boldsymbol{\nabla} \times \boldsymbol{\nabla} \times \mathbf{E}_v - \frac{\omega^2}{c^2}\mathbf{E}_v = 0 \\
\Rightarrow \boldsymbol{\nabla} \times \boldsymbol{\nabla} \times \mathbf{E}_v^* = \frac{\omega^{*2}}{c^2}\mathbf{E}_v^*
\end{aligned}$$

over a source-free vacuum region, leading to

$$\mathbf{H}_v^* \cdot \mathbf{H}_s = \frac{1}{\mu_0^2|\omega|^2}\boldsymbol{\nabla} \boldsymbol{\cdot} (\mathbf{E}_s \times \boldsymbol{\nabla} \times \mathbf{E}_v^*) + \frac{\omega^*}{\mu_0^2 c^2 \omega}\mathbf{E}_s \cdot \mathbf{E}_v^*,$$

$$\begin{aligned}
i\omega\mu_0 \int_{V_k} \mathbf{H}_v^* \cdot \mathbf{H}_s\, \mathrm{d}V &= \frac{i}{\mu\omega^*}\int_{\partial V_k} \mathrm{d}\sigma \cdot (\mathbf{E}_s \times \boldsymbol{\nabla} \times \mathbf{E}_v^*) + i\omega^*\epsilon_0 \int_{V_k} \mathbf{E}_s \cdot \mathbf{E}_v^*\, \mathrm{d}V \\
&= \int_{\partial V_k} \mathrm{d}\sigma \cdot (\mathbf{E}_s \times \mathbf{H}_v^*) + i\omega^*\epsilon_0 \int_{V_k} \mathbf{E}_s \cdot \mathbf{E}_v^*\, \mathrm{d}V.
\end{aligned}$$

Thus we have the useful relation

$$i\omega\mu_0 \int_{V_k} \mathbf{H}_v^* \cdot \mathbf{H}_s\, \mathrm{d}V - \int_{\partial V_k} \mathrm{d}\sigma \cdot (\mathbf{E}_s \times \mathbf{H}_v^*) - i\omega^*\epsilon_0 \int_{V_k} \mathbf{E}_s \cdot (\mathbb{I} + \mathbb{I}_s\overline{\chi}^*\mathbb{I}_s) \cdot \mathbf{E}_v^*\, \mathrm{d}V = -i\omega^*\epsilon_0 \int_{V_k} \mathbf{E}_s \cdot \mathbb{I}_s\overline{\chi}^*\mathbb{I}_s \cdot \mathbf{E}_v^*\, \mathrm{d}V. \tag{S11}$$

Similarly the other cross terms satisfy the relation

$$\begin{aligned}
&i\omega\mu_0 \int_{V_k} \mathbf{H}_v^* \cdot \mathbf{H}_s\, \mathrm{d}V - \int_{\partial V_k} \mathrm{d}\sigma \cdot (\mathbf{E}_s \times \mathbf{H}_v^*) - i\omega^*\epsilon_0 \int_{V_k} \mathbf{E}_s \cdot (\mathbb{I} + \mathbb{I}_s\overline{\chi}^*\mathbb{I}_s) \cdot \mathbf{E}_v^*\, \mathrm{d}V \\
&= -i\omega^*\epsilon_0 \int_{V_k} \mathbf{E}_s \cdot \mathbb{I}_s\overline{\chi}^*\mathbb{I}_s \cdot \mathbf{E}_v^*\, \mathrm{d}V + \int_{V_k} \mathbf{E}_v \cdot \mathbf{J}_s^*\, \mathrm{d}V
\end{aligned} \tag{S12}$$

where the additional $\int_{V_k} \mathbf{E}_v \cdot \mathbf{J}_s^* \, \mathrm{d}V$ comes from from $\mathbf{E}_s$ satisfying a vector wave equation with the polarization currents as source:

$$\boldsymbol{\nabla} \times \boldsymbol{\nabla} \times \mathbf{E}_s - \frac{\omega^2}{c^2}\mathbf{E}_s = i\omega\mu_0 \mathbf{J}_s.$$

Substituting these cross-term relations into the expression and taking the complex conjugate gives

$$\int_{V_k} \mathbf{E}_v^* \cdot \mathbf{J}_s \, \mathrm{d}V = -i\omega\epsilon_0 \int_{V_k} \mathbf{E}_t^* \cdot \mathbb{I}_s \overline{\chi} \mathbb{I}_s \cdot \mathbf{E}_t \, \mathrm{d}V - \int_{V_k} \mathbf{E}_s^* \cdot \mathbf{J}_s \, \mathrm{d}V. \tag{S13}$$

At this point, we can replace the polarization current $\mathbf{J}_s$, scattered electric field E_s, and total electric field E_t with the polarization density $\mathbf{P}$ via the following relations

$$\mathbf{J}_s = -i\omega\mathbf{P} \qquad \mathbf{P} = \epsilon_0 \overline{\chi} \mathbb{I}_s \cdot \mathbf{E}_t \qquad \mathbf{E}_s = \frac{1}{\epsilon_0}\mathbb{G} \cdot \mathbf{P} \tag{S14}$$

to finally obtain

$$\int_{V_k} \mathbf{E}_v \cdot \left(\frac{\mathbf{P}}{\epsilon_0}\right) \mathrm{d}V = \int_{V_k} \left(\frac{\mathbf{P}}{\epsilon_0}\right)^* \cdot \left(\frac{1}{\chi^*} - \mathbb{G}^*\right) \cdot \left(\frac{\mathbf{P}}{\epsilon_0}\right) \mathrm{d}V. \tag{S15}$$

8 Lagrangian duality given global constraints

In this section we detail how the dual problem for the two global power conservation constraints is equivalent to the dual problem for a single constraint with an additional parameter in the form of a complex phase rotation.

We are interested in placing dual bounds on the power extracted from a dipole source given global conservation of power:

$$\text{maximize} \quad \rho_{sca} = -\frac{1}{2}\,\mathrm{Im}\{\tilde{\omega}\,\langle \mathbf{E}_i^* | \mathbf{P} \rangle\} \tag{S16a}$$

$$\text{such that} \quad \mathrm{Re}\,\langle \mathbf{E}_v | \mathbf{P} \rangle - \langle \mathbf{P} | \mathrm{Sym}\mathbb{U} | \mathbf{P} \rangle = 0 \tag{S16b}$$

$$\mathrm{Im}\,\langle \mathbf{E}_v | \mathbf{P} \rangle - \langle \mathbf{P} | \mathrm{Asym}\mathbb{U} | \mathbf{P} \rangle = 0. \tag{S16c}$$

The Lagrangian for this constrained optimization problem is

$$\begin{aligned}
\mathcal{L}(\mathbf{P}, \alpha_{Re}, \alpha_{Im}) &= -\frac{1}{2}\,\mathrm{Im}\{\tilde{\omega}\,\langle \mathbf{E}_i^* | \mathbf{P} \rangle\} + \alpha_{Re}\big[\,\mathrm{Re}\,\langle \mathbf{E}_v | \mathbf{P} \rangle - \langle \mathbf{P} | \mathrm{Sym}\mathbb{U} | \mathbf{P} \rangle\,\big] + \alpha_{Im}\big[\,\mathrm{Im}\,\langle \mathbf{E}_v | \mathbf{P} \rangle - \langle \mathbf{P} | \mathrm{Asym}\mathbb{U} | \mathbf{P} \rangle\,\big] \\
&= -\frac{1}{2}\,\mathrm{Im}\{\tilde{\omega}\,\langle \mathbf{E}_i^* | \mathbf{P} \rangle\} + \big(\frac{\alpha_{Re}}{2} + \frac{\alpha_{Im}}{2i}\big)\,\langle \mathbf{E}_v | \mathbf{P} \rangle + \big(\frac{\alpha_{Re}}{2} - \frac{\alpha_{Im}}{2i}\big)\,\langle \mathbf{P} | \mathbf{E}_v \rangle \\
&+ \langle \mathbf{P} | \big[\big(\frac{\alpha_{Re}}{2} + \frac{\alpha_{Im}}{2i}\big)\mathbb{U} + \big(\frac{\alpha_{Re}}{2} - \frac{\alpha_{Im}}{2i}\big)\mathbb{U}^\dagger\big] | \mathbf{P} \rangle\,,
\end{aligned}$$

With the corresponding dual function

$$\mathcal{D}(\alpha_{Re}, \alpha_{Im}) = \max_{\mathbf{P}} \mathcal{L}(\mathbf{P}, \alpha_{Re}, \alpha_{Im}). \tag{S17}$$

Now define $\alpha \equiv \sqrt{\alpha_{Re}^2 + \alpha_{Im}^2}$ and a complex phase rotation $p \equiv e^{i\theta} = (\alpha_{Im} + i\alpha_{Re})/\alpha$ the Lagrangian can be re-written as

$$\begin{aligned}
\mathcal{L}(\mathbf{P}, \alpha; \theta) &= -\frac{1}{2}\,\mathrm{Im}\{\tilde{\omega}\,\langle \mathbf{E}_i^* | \mathbf{P} \rangle\} + \alpha\Bigg(\frac{e^{i\theta}\,\langle \mathbf{E}_i^* | \mathbf{P} \rangle - e^{-i\theta}\,\langle \mathbf{P} | \mathbf{E}_i^* \rangle}{2i} - \langle \mathbf{P} | \frac{e^{i\theta}\mathbb{U} - e^{-i\theta}\mathbb{U}^\dagger}{2i} | \mathbf{P} \rangle\Bigg) \\
&= -\frac{1}{2}\,\mathrm{Im}\{\tilde{\omega}\,\langle \mathbf{E}_i^* | \mathbf{P} \rangle\} + \alpha\big[\,\mathrm{Im}(p\,\langle \mathbf{E}_i^* | \mathbf{P} \rangle) - \langle \mathbf{P} | \mathrm{Asym}(p\mathbb{U}) | \mathbf{P} \rangle\,\big]
\end{aligned} \tag{S18}$$

which is exactly the Lagrangian of the single constraint optimization

$$\text{maximize} \quad \rho_{sca} = -\frac{1}{2}\,\mathrm{Im}\{\tilde{\omega}\,\langle \mathbf{E}_i^* | \mathbf{P} \rangle\} \tag{S19a}$$

$$\text{such that} \quad \mathrm{Im}(p\,\langle \mathbf{E}_v | \mathbf{P} \rangle) - \langle \mathbf{P} | \mathrm{Asym}(p\mathbb{U}) | \mathbf{P} \rangle = 0 \tag{S19b}$$

with corresponding dual function

$$\mathcal{D}(\alpha;\theta) = \max_{\mathbf{P}} \mathcal{L}(\mathbf{P},\alpha;\theta). \tag{S20}$$

Now it is clear that $(e^{i\theta},\alpha)$ is just an alternate parametrization of the multiplier space of $(\alpha_{Re},\alpha_{Im})$, hence the tightest dual bound is

$$\min_{\alpha_{Re},\alpha_{Im}} \mathcal{D}(\alpha_{Re},\alpha_{Im}) = \min_{\theta}\min_{\alpha} \mathcal{D}(\alpha;\theta). \tag{S21}$$

We can now derive an expression for $\min_\alpha \mathcal{D}(\alpha;\theta)$ for fixed phase rotation θ. First, note that the Lagrangian $\mathcal{L}(\mathbf{P},\alpha;\theta)$ only has a finite maximum when $\mathrm{Asym}(p\mathbb{U}) \succ 0$. See the following section for a spectral analysis of $\mathrm{Asym}(p\mathbb{U})$ ascertaining the existence and range of complex phase rotation p such that $\mathrm{Asym}(p\mathbb{U}) \succ 0$. We evaluate (S20) by calculating the stationary point of $\mathcal{L}$ with respect to $|\mathbf{P}\rangle$:

$$\frac{\partial \mathcal{L}}{\partial \langle \mathbf{P}|} = 0 \quad \Rightarrow \quad |\mathbf{P}\rangle = -\frac{i\tilde{\omega}^*}{4\alpha}\mathrm{Asym}(p\mathbb{U})^{-1}\,|\mathbf{E}_v^*\rangle + \frac{i}{2}p^*\mathrm{Asym}(p\mathbb{U})^{-1}\,|\mathbf{E}_v\rangle\,. \tag{S22}$$

This stationary point maximizes $\mathcal{L}$ when $\mathrm{Asym}(p\mathbb{U}) \succ 0$. Given this positive definite condition, the primal problem (S19) is also a convex problem with a non-empty feasible set, so strong duality holds [cite]. Thus to solve for the optimal $\bar{\alpha}$ that minimizes $\mathcal{D}(\alpha;\theta)$, we substitute (S22) into (S19b) and obtain

$$\bar{\alpha} = \sqrt{\frac{|\tilde{\omega}|^2}{4|p|^2}\frac{\langle \mathbf{E}_v^* | \mathrm{Asym}(p\mathbb{U})^{-1} | \mathbf{E}_v^* \rangle}{\langle \mathbf{E}_v | \mathrm{Asym}(p\mathbb{U})^{-1} | \mathbf{E}_v \rangle}}. \tag{S23}$$

Now (S22) and (S23) can be simultaneously substituted back into (S20) to get the bound

$$\rho_{sca} \le \min_{\alpha} \mathcal{D}(\alpha;\theta) = \frac{|\tilde{\omega}|}{4}\sqrt{\langle \mathbf{E}_v^* | \mathrm{Asym}(p\mathbb{U})^{-1} | \mathbf{E}_v^* \rangle\;\langle \mathbf{E}_v | \mathrm{Asym}(p\mathbb{U})^{-1} | \mathbf{E}_v \rangle} \\ - \frac{1}{4}\,\mathrm{Re}\left\{ p^*\tilde{\omega}\,\langle \mathbf{E}_v^* |\, \mathrm{Asym}(p\mathbb{U})^{-1}\,| \mathbf{E}_v \rangle \right\} \tag{S24}$$

as seen in the main text.

9 Spectral analysis of Green's function

Notation: in this and future sections of the supplementary info we will sometimes use the spatial wavevector $\tilde{k} = \tilde{\omega}/c = \tilde{\omega}$; in the main text only $\tilde{\omega}$ is used to reduce the number of different variables shown and to take advantage of the dimensionless units $c = 1$.

Starting from the planewave expansion of $\mathbb{G}$ as given by [6] (multiplied by $\tilde{k}^2$ following our convention)

$$\mathbb{G} = \frac{1}{(2\pi)^3}\iiint \mathrm{d}^3\mathbf{k}\, e^{i\mathbf{k}\cdot(\mathbf{r}-\mathbf{r}')}\frac{\mathbb{I}\tilde{k}^2 - \mathbf{k}\otimes\mathbf{k}}{k^2 - \tilde{k}^2}, \tag{S25}$$

we have

$$\mathrm{Asym}\mathbb{G} = \frac{1}{(2\pi)^3} \iiint \mathrm{d}^3\mathbf{k} e^{i\mathbf{k}\cdot(\mathbf{r}-\mathbf{r}')} \left\{ \mathrm{Im}\left(\frac{\tilde{k}^2}{k^2-\tilde{k}^2}\right)\mathbb{I} - \mathrm{Im}\left(\frac{k^2}{k^2-\tilde{k}^2}\right)\hat{\mathbf{k}}\otimes\hat{\mathbf{k}} \right\}. \tag{S26}$$

Now $\tilde{k}^2 = \tilde{k}_r^2 - \tilde{k}_i^2 + 2i\tilde{k}_r\tilde{k}_i$; for notational convenience define $A_r \equiv \mathrm{Re}\left\{\tilde{k}^2\right\} = \tilde{k}_r^2 - \tilde{k}_i^2$ and $A_i = \mathrm{Im}\left\{\tilde{k}^2\right\} = 2\tilde{k}_r\tilde{k}_i$. Taking the imaginary part, we get

$$\mathrm{Asym}\mathbb{G} = \frac{1}{(2\pi)^3} \iiint \mathrm{d}^3\mathbf{k} e^{i\mathbf{k}\cdot(\mathbf{r}-\mathbf{r}')} \left\{ \frac{A_i k^2}{(k^2-A_r)^2+A_i^2}\mathbb{I} - \frac{A_i k^2}{(k^2-A_r)^2+A_i^2}\hat{\mathbf{k}}\otimes\hat{\mathbf{k}} \right\}. \tag{S27}$$

It is apparent now that the eigenwaves of $\mathrm{Asym}\mathbb{G}$ have the form $\hat{\mathbf{e}}e^{i\mathbf{k}\cdot\mathbf{r}}$, where $\hat{\mathbf{e}}$ is an eigenvector of the 3×3 matrix in parentheses. Under a triad that includes $\hat{\mathbf{k}}$, the 3×3 matrix is diagonal, and we see the longitudinal eigenwaves $\hat{\mathbf{k}}e^{i\mathbf{k}\cdot\mathbf{r}}$ have eigenvalue 0. The transverse eigenwaves have eigenvalues

$$\rho_t(k) = \frac{A_i k^2}{(k^2-A_r)^2+A_i^2} = \frac{2\tilde{k}_r\tilde{k}_i k^2}{(k^2-\tilde{k}_r^2+\tilde{k}_i^2)^2+(2\tilde{k}_r\tilde{k}_i)^2} \geq 0. \tag{S28}$$

Some observations about the scaling of these eigenvalues that are relevant for later discussion:

- $\mathrm{Asym}\mathbb{G}$ is positive semi-definite. The null-space always includes the longitudinal waves (which are irrelevant to the TM case). For single frequencies $\tilde{k}_i = 0$ the null-space includes all evanescent waves; for finite bandwidth $\tilde{k}_i > 0$ the null-space only contains extremely fast oscillations / extreme evanescent waves in the limit $|k| \to \infty$.
- Some idea of the scaling of $\mathrm{Asym}\mathbb{G}^{-1}$ can be seen through the inverse of the transverse eigenvalues $1/\rho_t = \frac{1}{A_i}k^2 + (\frac{A_r^2}{A_i} + A_i)\frac{1}{k^2} - 2\frac{A_r}{A_i}$, which for $k \geq \tilde{k}_r$ has a minimum around $k = |\tilde{k}|$ before scaling $\sim k^2$ as $k \to \infty$.

10 Spectral Analysis of Asym$(p\mathbb{U})$

We have $\mathrm{Asym}(p\mathbb{U}) = \mathrm{Asym}(\chi^{-1\dagger}p) + \mathrm{Asym}(p^*\mathbb{G})$. $\mathrm{Asym}(\chi^{-1\dagger}p)$ contributes a constant to all eigenvalues; we focus our attention on the second term $\mathrm{Asym}(p^*\mathbb{G})$. Similar to the analysis in the previous subsection, we have

$$\mathrm{Asym}\mathbb{G} = \frac{1}{(2\pi)^3} \iiint \mathrm{d}^3\mathbf{k} e^{i\mathbf{k}\cdot(\mathbf{r}-\mathbf{r}')} \left\{ \mathrm{Im}\left(\frac{p^*\tilde{k}^2}{k^2-\tilde{k}^2}\right)\mathbb{I} - \mathrm{Im}\left(\frac{p^*k^2}{k^2-\tilde{k}^2}\right)\hat{\mathbf{k}}\otimes\hat{\mathbf{k}} \right\}. \tag{S29}$$

The longitudinal and transverse eigenvalues of $\mathrm{Asym}(p^*\mathbb{G})$ come out to be

$$\rho_{\mathbb{G},l}(p) = p_i,$$
$$\rho_{\mathbb{G},t}(p) = \frac{p_r k^2 A_i + p_i[-A_r(k^2-A_r)+A_i^2]}{(k^2-Ar)^2+A_i^2}.$$

The eigenvalues of $\mathrm{Asym}(p\mathbb{U})$ are

$$\rho_{\mathbb{U},l} = \frac{\chi_i}{\chi_r^2+\chi_i^2}p_r + \left(1+\frac{\chi_r}{\chi_r^2+\chi_i^2}\right)p_i, \tag{S30}$$

$$\rho_{\mathbb{U},t} = \frac{p_r\chi_i + p_i\chi_r}{\chi_r^2+\chi_i^2} + \frac{p_r k^2 A_i + p_i[-A_r(k^2-A_r)+A_i^2]}{(k^2-Ar)^2+A_i^2}. \tag{S31}$$

In order for the dual bound (S24) to be valid we need $\mathrm{Asym}(p\mathbb{U})$ to be positive definite, i.e., $\rho_{\mathbb{U}} > 0$.

10.1 Lossless dielectrics

For lossless dielectrics we have $\chi_i = 0$, $\chi_r > 0$. The PD condition $\rho_{\mathbb{U},l} > 0$ then leads to $p_i > 0$. Setting $p_r = 1$, we require

$$\rho_{\mathbb{U},t} = \frac{1}{\chi_r} p_i + \frac{k^2 A_i + p_i[-A_r(k^2 - A_r) + A_i^2]}{(k^2 - A_r)^2 + A_i^2} > 0. \tag{S32}$$

Defining for convenience $u \equiv k^2$, the problem is then finding

$$\min_{u \geq 0} f(u) = \frac{(A_i - p_i A_r)u + p_i(A_r^2 + A_i^2)}{(u - A_r)^2 + A_i^2}. \tag{S33}$$

It is clear that $f(0) = p_i > 0$ and $\lim_{u\to\infty} f(u) = 0$. In the special case $p_i = A_i/A_r$, the only critical point is $u^* = A_r$ which is a maximum, so $\min f(u) = 0$ and $\rho_{\mathbb{U},t} = \frac{1}{\chi_r} p_i > 0$. When $p_i \neq A_i/A_r$ the critical points are (assuming $A_i \ll A_r$)

$$u^{*\pm} \approx \frac{p_i A_r^2 \pm \sqrt{1 + p_i^2} A_i A_r}{p_i A_r - A_i}. \tag{S34}$$

If $p_i < A_i/A_r$ then $u^{*+} < 0$ and irrelevant, while u^{*-} is a maximum, so $\min f(u) = 0$. If $p_i > A_i/A_r$, then u^{*+} is the minimum, with

$$\min f(u) = f(u^{*+}) \approx \frac{-\sqrt{1 + p_i^2} A_r A_i}{\left[\frac{(1+\sqrt{1+p_i^2})A_r A_i}{p_i A_r - A_i}\right]^2 + A_i^2} < 0. \tag{S35}$$

Thus for lossless dielectrics, $\mathrm{Asym}(p\mathbb{U})$ is PD given $p_r = 1$ and $0 < p_i \leq A_i/A_r$. For specific values of χ_r, larger rotation angles with $p_i > A_i/A_r$ is possible. By how much? Set

$$p_i = c \cdot \frac{A_i}{A_r}; \tag{S36}$$

now, assume that $p_i \ll 1$ in the limit $A_i \to 0$ for vanishing bandwidth, this assumption can be checked for consistency at the end. This yields

$$\min f(u) \approx -\frac{(c-1)^2}{4} \frac{A_i}{A_r}. \tag{S37}$$

Then

$$\rho_{\mathbb{U},t} > 0 \Rightarrow \frac{p_i}{\chi_r} > \frac{(c-1)^2}{4} \frac{A_i}{A_r} \Rightarrow c^2 - \left(\frac{4}{\chi_r} + 2\right) c + 1 < 0. \tag{S38}$$

Conclude that

$$c < 1 + 2\left[\frac{1}{\chi_r} + \sqrt{\frac{1}{\chi_r^2} + \frac{1}{\chi_r}}\right], \tag{S39}$$

so c_{max} is a monotonic function of χ_r and not dependent on the bandwidth, $p_{i,max} \propto A_i \ll 1$ in the limit $A_i \to 0$.

11 Global constraint bounds for 2D TM dipole near a half-space

In this section we evaluate (S24) explicitly for the case of 2D TM dipole source $\mathbf{J}(x', y') = \delta(x')\delta(y' + d)$ a distance d away from a half-space design region $V = \{(x, y)|y > 0\}$.

Notation: in the main text the $\|$ and $\perp$ symbols were used to indicate directions parallel and perpendicular to the surface of the half-space design region, respectively. In this section we use explicit Cartesian coordinates with x being $\|$ and y being $\perp$.

11.1 TM Green's function and dipole field

Following [6], the 2D TM Green's function is

$$\mathbb{G}^{TM}(\rho, \rho') = \tilde{k}^2 \frac{i}{4\pi} \begin{cases} \int_{-\infty}^{\infty} \mathrm{d}k_x \frac{\hat{z}\hat{z}}{k_y} e^{ik_x(x-x')} e^{ik_y(y-y')} & y > y' \\ \int_{-\infty}^{\infty} \mathrm{d}k_x \frac{\hat{z}\hat{z}}{k_y} e^{ik_x(x-x')} e^{-ik_y(y-y')} & y < y' \end{cases} \tag{S40}$$

where $\tilde{k} = \tilde{k}_r + i\tilde{k}_i$ is complex for finite bandwidths, $k_y = \sqrt{\tilde{k}^2 - kx^2}$ always taking the root with positive imaginary part.

The dipole source $\mathbf{J}(x', y') = \delta(x')\delta(y' + d)$ produces a vacuum electric field within the $y > 0$ design region

$$\mathbf{E}_v^{TM}(x, y) = \int_{-\infty}^{\infty} \frac{e^{ik_x x}}{\sqrt{2\pi}} \mathbf{E}_{v,k_x}^{TM}(y)\, \mathrm{d}k_x, \tag{S41a}$$

$$\mathbf{E}_{v,k_x}^{TM}(y) = -\frac{\tilde{k}}{2\sqrt{2\pi}} \frac{1}{k_y} e^{ik_y y} e^{ik_y d}. \tag{S41b}$$

The single frequency vacuum dipole radiation is

$$\rho_0^{TM} = \frac{\mathrm{Re}\left\{\tilde{k}\right\}}{8} = \frac{\pi}{4\lambda_0}. \tag{S42}$$

The composite $\mathrm{Asym}(p\mathbb{U}^{TM})$ integral operator is

$$\mathrm{Asym}(p\mathbb{U}^{TM})(x, y, x', y') = \int_{-\infty}^{\infty} \mathrm{d}k_x \frac{e^{ik_x x}}{\sqrt{2\pi}} \mathrm{Asym}(p\mathbb{U}^{TM})_{k_x}(y, y') \frac{e^{-ik_x x'}}{\sqrt{2\pi}} \tag{S43a}$$

with

$$\mathrm{Asym}(p\mathbb{U}^{TM})_{k_x}(y, y') = \mathrm{Im}\{p/\chi^*\}\delta(y - y') + \frac{1}{2}\mathrm{Re}\left\{p^* \frac{\tilde{k}^2}{k_y} e^{ik_y|y-y'|}\right\}. \tag{S43b}$$

11.2 Simplification of (S24)

For the half-space design region, (S24) may be simplified by observing that $\left\langle \mathbf{E}_v^{TM} \middle| \mathrm{Asym}(p\mathbb{U}^{TM})^{-1} \middle| \mathbf{E}_v^{TM} \right\rangle = \left\langle \mathbf{E}_v^{TM*} \middle| \mathrm{Asym}(p\mathbb{U}^{TM})^{-1} \middle| \mathbf{E}_v^{TM*} \right\rangle$. We have

$$\left\langle \mathbf{E}_v^{TM} \middle| \mathrm{Asym}(p\mathbb{U}^{TM})^{-1} \middle| \mathbf{E}_v^{TM} \right\rangle = \frac{\left|\tilde{k}\right|^2}{8\pi} \int_{-\infty}^{\infty} \mathrm{d}k_x \frac{e^{-2k_{yi}d}}{\left|k_y\right|^2} \iint_0^{\infty} e^{-ik_y^* y} \mathrm{Asym}(p\mathbb{U}^{TM})_{k_x}^{-1}(y, y') e^{ik_y y}\, \mathrm{d}y' \mathrm{d}y \tag{S44}$$

where we have made multiple use of the Fourier completeness relation $\frac{1}{2\pi}\int_{-\infty}^{\infty} e^{i(k_x-k_x')x}\,\mathrm{d}x = \delta(k_x - k_x')$. Now,

$$\begin{aligned}\mathbf{E}_v^{TM*}(x,y) &= -\frac{\tilde{k}^*}{2\sqrt{2\pi}}\int_{-\infty}^{\infty}\frac{e^{-ik_x x}}{\sqrt{2\pi}}\frac{1}{k_y^*}e^{-ik_y^* y}e^{-ik_y^* d}\,\mathrm{d}k_x \\ &= -\frac{\tilde{k}^*}{2\sqrt{2\pi}}\int_{-\infty}^{\infty}\frac{e^{ik_x x}}{\sqrt{2\pi}}\frac{1}{k_y^*}e^{-ik_y^* y}e^{-ik_y^* d}\,\mathrm{d}k_x \qquad kx \to -kx \end{aligned} \tag{S45}$$

where in the second line we have made use of the fact that $k_y = \sqrt{\tilde{k}^2 - k_x^2}$ is invariant to the sign of k_x. This gives

$$\left\langle \mathbf{E}_v^{TM}\middle|\mathrm{Asym}(p\mathbb{U}^{TM})^{-1}\middle|\mathbf{E}_v^{TM}\right\rangle = \frac{\left|\tilde{k}\right|^2}{8\pi}\int_{-\infty}^{\infty}\mathrm{d}k_x\frac{e^{-2k_{yi}d}}{\left|k_y\right|^2}\iint_0^{\infty} e^{ik_y y}\mathrm{Asym}(p\mathbb{U}^{TM})_{k_x}^{-1}(y,y')e^{-ik_y^* y}\,\mathrm{d}y'\mathrm{d}y. \tag{S46}$$

Now from (S43b) we see that $\mathrm{Asym}(p\mathbb{U}^{TM})_{k_x}(y,y')$ is invariant under exchange of the y, y' dummy integration variables. Since (S44) and (S46) are related by the exchange of y, y', conclude that

$$\left\langle \mathbf{E}_v^{TM}\middle|\mathrm{Asym}(p\mathbb{U}^{TM})^{-1}\middle|\mathbf{E}_v^{TM}\right\rangle = \left\langle \mathbf{E}_v^{TM*}\middle|\mathrm{Asym}(p\mathbb{U}^{TM})^{-1}\middle|\mathbf{E}_v^{TM*}\right\rangle. \tag{S47}$$

This allows us to simplify (S24) to

$$\rho_{sca}^{TM} \le \frac{|\tilde{\omega}|}{4}\left\langle \mathbf{E}_v^{TM}\middle|\mathrm{Asym}(p\mathbb{U}^{TM})^{-1}\middle|\mathbf{E}_v^{TM}\right\rangle - \frac{1}{4}\mathrm{Re}\left\{p^*\tilde{\omega}\left\langle \mathbf{E}_v^{TM*}\right|\mathrm{Asym}(p\mathbb{U})^{-1}\left|\mathbf{E}_v^{TM}\right\rangle\right\}. \tag{S48}$$

For certain asymptotic and approximation analyses, we will also make use of (S47) and the Cauchy-Schwartz inequality to relax the second term in (S48), yielding

$$\rho_{sca}^{TM} \le \frac{|\tilde{\omega}|}{2}\left\langle \mathbf{E}_v^{TM}\middle|\mathrm{Asym}(p\mathbb{U}^{TM})^{-1}\middle|\mathbf{E}_v^{TM}\right\rangle \tag{S49}$$

11.3 Evaluation of $\mathrm{Asym}(p\mathbb{U}^{TM})^{-1}\left|\mathbf{E}_v^{TM}\right\rangle$

The core calculation in (S48) is the evaluation of

$$\mathrm{Asym}(p\mathbb{U}^{TM})^{-1}\left|\mathbf{E}_v^{TM}\right\rangle = -\frac{\tilde{k}}{2\sqrt{2\pi}}\int_{-\infty}^{\infty}\mathrm{d}k_x\frac{e^{ik_x x}}{\sqrt{2\pi}}\frac{e^{ik_y d}}{k_y}\int_0^{\infty}\mathrm{Asym}(p\mathbb{U}^{TM})_{k_x}^{-1}(y,y')e^{ik_y y'}\,\mathrm{d}y'. \tag{S50}$$

Defining

$$f(y) = \int_0^{\infty}\mathrm{Asym}(p\mathbb{U}^{TM})_{k_x}^{-1}(y,y')e^{ik_y y'}\,\mathrm{d}y', \tag{S51}$$

we have

$$\int_0^{\infty}\mathrm{Asym}(p\mathbb{U}^{TM})_{k_x}(y,y')f(y')\,\mathrm{d}y' = e^{ik_y y'}. \tag{S52}$$

The action of $\mathrm{Asym}(p\mathbb{U})_{k_x}$ is a convolution over y; this suggests that the Laplace transform $\mathscr{L}\{h(t)\}(s) = \int_0^{\infty} h(t)e^{-st}\,\mathrm{d}t$ may be used to solve (S52). We now take the Laplace transform of both sides of (S52), defining $F(s) \equiv \mathscr{L}\{f(y)\}$ and the auxiliary variable $B \equiv p^*\frac{\tilde{k}^2}{k_y}$. The LHS gives

$$\begin{aligned}\mathrm{Im}\left\{\frac{p}{\chi^*}\right\}F(s) &+ \frac{B_r+iB_i}{4}\mathscr{L}\left\{\int_0^y e^{-(k_{yi}-ik_{yr})(y-y')}f(y')\,\mathrm{d}y'\right\} + \frac{B_r-iB_i}{4}\mathscr{L}\left\{\int_0^y e^{-(k_{yi}+ik_{yr})(y-y')}f(y')\,\mathrm{d}u'\right\} \\ &+ \frac{B_r+iB_i}{4}\mathscr{L}\left\{\int_y^{\infty} e^{(k_{yi}-ik_{yr})(y-y')}f(y')\,\mathrm{d}y'\right\} + \frac{B_r-iB_i}{4}\mathscr{L}\left\{\int_y^{\infty} e^{(k_{yi}+k_{yr})(y-y')}f(y')\,\mathrm{d}y'\right\}.\end{aligned}$$

The RHS is simply

$$\mathscr{L}\left\{e^{(-k_{yi}+ik_{yr})y}\right\} = \frac{1}{s+(k_{yi}-ik_{yr})}.$$

Evaluating the LHS, the Laplace transform of a convolution gives

$$\mathscr{L}\left\{\int_0^y e^{-a(y-y')}f(y')\,\mathrm{d}y'\right\} = \frac{F(s)}{s+a}. \tag{S53}$$

The transform of the other type of integral can be evaluated explicitly to a simple form:

$$\begin{aligned}
\mathscr{L}\left\{\int_y^\infty e^{a(y-y')}f(y')\,\mathrm{d}y'\right\} &= \int_0^\infty e^{-sy}\int_y^\infty e^{a(y-y')}f(y')\,\mathrm{d}y'\,\mathrm{d}y \\
&= \int_0^\infty e^{-ay'}f(y')\left(\int_0^{y'} e^{-(s-a)y}\,\mathrm{d}y\right)\mathrm{d}y' \qquad \text{exchange order of integration} \\
&= \int_0^\infty f(y')e^{-ay'}\frac{1}{a-s}\left(e^{(a-s)y'}-1\right)\mathrm{d}y' \\
&= \frac{1}{a-s}\left(\int_0^\infty f(y')e^{-sy'}\,\mathrm{d}y' - \int_0^\infty f(y')e^{-ay'}\,\mathrm{d}y'\right) \\
&= \frac{1}{a-s}(F(s)-F(a)).
\end{aligned} \tag{S54}$$

Thus $F(s)$ satisfies

$$\begin{aligned}
\frac{1}{s+(k_{yi}-ik_{yr})} = \frac{p_i}{\chi}Fs) &+ \frac{Br+iB_i}{4}\frac{F(s)}{s+(k_{yi}-ik_{yr})} \\
&+ \frac{B_r-iB_i}{4}\frac{F(s)}{s+(k_{yi}+ik_{yr})} \\
&+ \frac{B_r+iB_i}{4}\frac{1}{(k_{yi}-ik_{yr})-s}\Big[F(s)-F(k_{yi}-ik_{yr})\Big] \\
&+ \frac{B_r-iB_i}{4}\frac{1}{(k_{yi}+ik_{yr})-s}\Big[F(s)-F(k_{yi}+ik_{yr})\Big].
\end{aligned} \tag{S55}$$

Solving this will give us an explicit form of $F(s)$ dependent on two free parameters $F(k_{yi}-ik_{yr})$ and $F(k_{yi}+ik_{yr})$. This seemingly gives, instead of a single solution $f(y)$, a whole family of solutions. However, as we shall see, only one member of this family belongs to $L^2[0,\infty)$, decaying as $y\to\infty$. The other members of this family grow exponentially as $y\to\infty$ which violates the requirements of Fubini's theorem [4] needed to justify the order of integration exchange used in (S54), and are not true solutions to (S52).

Solving for $F(s)$ yields

$$F(s) = \frac{F_{num}(s)}{F_{denom}(s)} \tag{S56a}$$

where the numerator is

$$\begin{aligned}
F_{num}(s) = [s-(k_{yi}-ik_{yr})][s^2-(k_{yi}+ik_{yr})^2] &- \frac{Br+iB_i}{4}\gamma_-[s+(k_{yi}-ik_{yr})][s^2-(k_{yi}+ik_{yr})^2] \\
&- \frac{B_r-iB_i}{4}\gamma_+[s+(k_{yi}+ik_{yr})][s^2-(k_{yi}-ik_{yr})^2]
\end{aligned} \tag{S56b}$$

and the denominator is

$$F_{denom}(s) = \mathrm{Im}\left\{\frac{p}{\chi^*}\right\}s^4 - \left[2\,\mathrm{Im}\left\{\frac{p}{\chi^*}\right\}(k_{yi}^2 - k_{yr}^2) + (B_r k_{yi} + B_i k_{yr})\right]s^2 + \mathrm{Im}\left\{\frac{p}{\chi^*}\right\}|k_y|^4 + (B_r k_{yi} - B_i k_{yr})|k_y|^2, \tag{S56c}$$

where for notational convenience, we have defined $\gamma_\pm \equiv F(k_{yi} \pm ik_{yr})$.

The roots r of $F_{denom}(s)$ are then the poles of $F(s)$:

$$(+,-)r_+ = (+,-)\left\{(k_{yi}^2 - k_{yr}^2) + \frac{1}{2\,\mathrm{Im}\{p/\chi^*\}}\left[(B_r k_{yi} + B_i k_{yr}) + \sqrt{\Delta}\right]\right\}^{1/2}, \tag{S57a}$$

$$(+,-)r_- = (+,-)\left\{(k_{yi}^2 - k_{yr}^2) + \frac{1}{2\,\mathrm{Im}\{p/\chi^*\}}\left[(B_r k_{yi} + B_i k_{yr}) - \sqrt{\Delta}\right]\right\}^{1/2}, \tag{S57b}$$

$$\Delta = (B_r k_{yi} + B_i k_{yr})^2 + 8\,\mathrm{Im}\left\{\frac{p}{\chi^*}\right\}\left(B_i k_{yr} k_{yi}^2 - B_r k_{yr}^2 k_{yi} - \mathrm{Im}\left\{\frac{p}{\chi^*}\right\}k_{yr}^2 k_{yi}^2\right), \tag{S57c}$$

$$F_{denom}(s) = \mathrm{Im}\left\{\frac{p}{\chi^*}\right\}(s - r_+)(s + r_+)(s - r_-)(s + r_-). \tag{S57d}$$

We see that the poles come in two pairs $\pm r_+$ and $\pm r_-$. In general, one each from $(+,-)r_+$ and $(+,-)r_-$ will have a positive real part and its opposite sign counterpart will have a negative real part; we take r_+ and r_- to be the poles with positive real parts (in the main text these are r_1 and r_2 respectively. The inverse Laplace transform is

$$f(y) = \mathscr{L}^{-1}\left\{F(s)\right\} = \frac{1}{2\pi i}\int_{T-i\infty}^{T+i\infty} F(s)e^{sy}\,\mathrm{d}s \tag{S58}$$

where $T \in \mathbb{R}$ is greater than the real parts of all the poles of $F(s)$. Since $y > 0$, we can deform the line integration and complete the contour in the $\mathrm{Re}\{s\} < 0$ half plane upon which the contour completion part decays to 0; thus the inverse Laplace transform picks out the residue of $F(s)e^{sy}$. It is clear then that the contribution to the residue from r_+ and r_- leads to functions with exponential growth as $y \to \infty$ whereas the contribution from $-r_+$ and $-r_+$ lead to functions with exponential decay as $y \to \infty$. To recover $f(y) \in L^2[0,\infty)$ we need to select free parameters $\gamma_\pm$ such that the residue for r_+ and r_- are 0. This leads to the following linear system of equations from which we solve for $\gamma_\pm$:

$$\begin{aligned}&[r_+ - (k_{yi} - ik_{yr})][r_+^2 - (k_{yi} + ik_{yr})^2] \\ &= \frac{B_r + iB_i}{4}[r_+ + (k_{yi} - ik_{yr})][r_+^2 - (k_{yi} + ik_{yr})^2]\gamma_- + \frac{Br - iB_i}{4}[r_+ + (k_{yi} + ik_{yr})][r_+^2 - (k_{yi} - ik_{yr})^2]\gamma_+,\end{aligned} \tag{S59a}$$

$$\begin{aligned}&[r_- - (k_{yi} - ik_{yr})][r_-^2 - (k_{yi} + ik_{yr})^2] \\ &= \frac{B_r + iB_i}{4}[r_- + (k_{yi} - ik_{yr})][r_-^2 - (k_{yi} + ik_{yr})^2]\gamma_- + \frac{Br - iB_i}{4}[r_- + (k_{yi} + ik_{yr})][r_-^2 - (k_{yi} - ik_{yr})^2]\gamma_+.\end{aligned} \tag{S59b}$$

$f(y)$ then takes on the form

$$f(y) = R_+e^{-r_+y} + R_-e^{-r_-y} \tag{S60}$$

with the coefficients of the exponentials (R_1 and R_2 in the main text) given by

$$
\begin{aligned}
R_\pm = \frac{1}{\mp 2\,\mathrm{Im}\{p/\chi^*\} r_\pm \cdot (r_+^2 - r_-^2)} \Big\{ & [-r_\pm - (k_{yi} - ik_{yr})][r_\pm^2 - (k_{yi} + ik_{yr})^2] \\
& - \frac{B_r + iB_i}{4} [-r_\pm + (k_{yi} - ik_{yr})][r_\pm^2 - (k_{yi} + ik_{yr})^2]\gamma_- \\
& - \frac{B_r - iB_i}{4} [-r_\pm + (k_{yi} + ik_{yr})][r_\pm^2 - (k_{yi} - ik_{yr})^2]\gamma_+ \Big\}.
\end{aligned} \tag{S61}
$$

(S60) can now be substituted back into (S50) and (S48) to get an analytical integral expression for the LDOS enhancement bound near a half-space:

$$
\rho_{sca} \leq \frac{1}{16\pi} \int_0^\infty \mathrm{Re}\left\{ -\frac{\tilde{k}^3 p\, e^{2ik_y d}}{k_y^2} \left(\frac{R_+}{r_+ - ik_y} + \frac{R_-}{r_- - ik_y} \right) + \frac{\left|\tilde{k}\right|^3 e^{-2k_{yi} d}}{|k_y|^2} \left(\frac{R_+}{r_+ + ik_y^*} + \frac{R_-}{r_- + ik_y^*} \right) \right\} \tag{S62}
$$

11.4 TE Green's function and dipole field

The 2D TE Green's function is

$$
\mathbb{G}^{TE}(x,y,x',y') = -\hat{y}\hat{y}\delta(x-x')\delta(y-y') + \tilde{k}^2 \frac{i}{4\pi} \begin{cases} \int_{-\infty}^{\infty} \mathrm{d}k_x \frac{\hat{h}(k_y)\hat{h}(k_y)}{k_y} e^{ik_x(x-x')} e^{ik_y(y-y')} & y > y' \\ \int_{-\infty}^{\infty} \mathrm{d}k_x \frac{\hat{h}(-k_y)\hat{h}(-k_y)}{k_y} e^{ik_x(x-x')} e^{-ik_y(y-y')} & y < y' \end{cases} \tag{S63}
$$

with the unit vectors $\hat{h}$ given by

$$
\hat{h}(k_y) = -\frac{k_y}{\tilde{k}}\hat{x} + \frac{k_x}{\tilde{k}}\hat{y} \qquad \hat{h}(-k_y) = \frac{k_y}{\tilde{k}}\hat{x} + \frac{k_x}{\tilde{k}}\hat{y}. \tag{S64}
$$

A dipole source of the form $\mathbf{J}^y(x',y') = \hat{y}\delta(x')\delta(y'+d)$ produces a vacuum field

$$
\mathbf{E}_v^{TEy}(x,y) = \int_{-\infty}^{\infty} \frac{e^{ik_x x}}{\sqrt{2\pi}} \mathbf{E}_{v,k_x}^{TEy}(y)\,\mathrm{d}k_x, \tag{S65a}
$$

$$
\mathbf{E}_{v,k_x}^{TEy}(y) = -\frac{\sqrt{2\pi}}{4\pi\tilde{k}} \cdot e^{ik_y d} e^{ik_y y} \cdot \left(-k_x \hat{x} + \frac{k_x^2}{k_y}\hat{y} \right). \tag{S65b}
$$

The vacuum dipole radiation is

$$
\rho_0^{TEy} = \frac{\mathrm{Re}\left\{\tilde{k}\right\}}{16} = \frac{\pi}{8\lambda_0}. \tag{S66}
$$

The composite $\mathrm{Asym}(p\mathbb{U}^{TE})$ integral operator is

$$
\begin{aligned}
\mathrm{Asym}(p\mathbb{U}^{TE}) = \int_{-\infty}^{\infty} \mathrm{d}k_x \frac{e^{ik_x(x-x')}}{2\pi} \Bigg\{ & \mathrm{Im}\left\{\frac{p}{\chi^*}\right\} \delta(y-y')(\hat{x}\hat{x} + \hat{y}\hat{y}) + \delta(y-y') p_i \hat{y}\hat{y} + \\
& \frac{1}{2}\mathrm{Re}\left\{p^* k_y e^{ik_y|y-y'|}\right\}\hat{x}\hat{x} + \frac{1}{2}\mathrm{Re}\left\{p^*(k_x^2/k_y) e^{ik_y|y-y'|}\right\}\hat{y}\hat{y} - \frac{1}{2}\mathrm{sgn}(y-y')\,\mathrm{Im}\left\{p^* k_x e^{ik_y|y-y'|}\right\}(\hat{x}\hat{y} + \hat{y}\hat{x}) \Bigg\}.
\end{aligned} \tag{S67}
$$

The corresponding global constraint bound for a TE dipole source near a half-space can be derived following an analogous procedure to that of a TM dipole source, though the expressions involved are tedious and was done in practice using Mathematica. For conciseness, the expressions are not reproduced here.

12 Asymptotic analysis

Based on the results from the prior Section, we can do asymptotic analysis to understand the observed scaling of the half-space bounds with regards to bandwidth, material, and separation.

12.1 TM bandwidth scaling

To understand the scaling of the LDOS bounds with bandwidth, it is illuminating to consider the contributions from the traveling waves and evanescent waves separately. Here traveling waves and evanescent waves refer to different k_x in the planewave decomposition of the dipole field: traveling waves have $|k_x| < \tilde{k}_r$ and a predominantly real k_y, propagating for long distances with decay rate proportional to the bandwidth $\tilde{k}_i$; evanescent waves have $|k_x| > \tilde{k}_r$ and a predominantly imaginary k_y, rapidly decaying along the y direction with decay rate strongly dependent on $|k_x|$.

12.1.1 Traveling wave contribution

We show that in the limit of zero bandwidth / single frequency, the contribution from just the traveling waves to the LDOS limits tends toward a finite constant. The following analysis is for TM fields but an analogous result can be obtained for TE fields.
We take the material susceptibility $|\chi| \to \infty$ and phase rotation $p = 1$ so the results are material-independent and $\text{Asym}(p\mathbb{U}^{TM}) = \text{Asym}\mathbb{G}^{TM}$:

$$\text{Asym}\mathbb{G}^{TM}(x, y; x', y') = \int_{-\infty}^{\infty} \frac{e^{ik_x(x-x')}}{2\pi} \text{Asym}\mathbb{G}^{TM}_{k_x}(y, y')\, \mathrm{d}k_x,$$

$$\text{Asym}\mathbb{G}^{TM}_{k_x}(y, y') = \frac{1}{2}\,\text{Re}\left\{ \frac{\tilde{k}^2}{k_y} e^{ik_y|y-y'|} \right\}.$$

In the limit of zero bandwidth / single frequency, $\tilde{k} \in \mathbb{R}$. Then for $|k_x| > \tilde{k}$, k_y is purely imaginary and $\text{Asym}\mathbb{G}^{TM}_{k_x} = 0$, leading to

$$\begin{aligned}\text{Asym}\mathbb{G}^{TM} &= \int_{-\tilde{k}}^{\tilde{k}} \mathrm{d}k_x \frac{e^{ik_x(x-x')}}{2\pi} \frac{\tilde{k}^2}{k_y} \cos k_y(y - y') \\ &= \int_{-\tilde{k}}^{\tilde{k}} \mathrm{d}k_x \frac{e^{ik_x(x-x')}}{2\pi} \frac{\tilde{k}^2}{k_y} \big[\cos(k_y y) \cos(k_y y') + \sin(k_y y) \sin(k_y y') \big].\end{aligned}$$

We consider a design region shaped as a slab with infinite extent in the x direction and a thickness h in the y direction with the origin at the center. This is different from a half-space, but as we shall see the final result is completely independent of h and thus should apply in the limit $h \to \infty$. The different parities of cos and sin about the origin allow us to directly write down the

eigenvectors of $\mathrm{Asym}\mathbb{G}_{k_x}$ as

$$|q_{cos}(k_y)\rangle = \frac{\cos(k_y y)}{a_{cos}}, \tag{S68a}$$

$$|q_{sin}(k_y)\rangle = \frac{\sin(k_y y)}{a_{sin}} \tag{S68b}$$

with normalization factor

$$a_{cos}^2 = \int_{-h/2}^{h/2} \cos^2(k_y y)\,\mathrm{d}y = \frac{1}{2k_y}(k_y h + \sin(k_y h)), \tag{S68c}$$

$$a_{sin}^2 = \int_{-h/2}^{h/2} \sin^2(k_y y)\,\mathrm{d}y = \frac{1}{2k_y}(k_y h - \sin(k_y h)) \tag{S68d}$$

and corresponding eigenvalues

$$\rho_{cos}(k_y) = \frac{\tilde{k}^2}{2k_y} a_{cos}^2, \tag{S68e}$$

$$\rho_{sin}(k_y) = \frac{\tilde{k}^2}{2k_y} a_{sin}^2. \tag{S68f}$$

We can thus write

$$\mathrm{Asym}\mathbb{G}^{TM} = \int_{-\tilde{k}}^{\tilde{k}} \mathrm{d}k_x \frac{e^{ik_x(x-x')}}{2\pi} \Big(\rho_{cos}(k_y)\,|q_{cos}(k_y)\rangle\,\langle q_{cos}(k_y)| + \rho_{sin}(k_y)\,|q_{sin}(k_y)\rangle\,\langle q_{sin}(k_y)| \Big). \tag{S69}$$

The dipole source $\mathbf{J}(x', y') = \delta(x')\delta(y' - d - h/2)$ generates an incident field with the traveling wave components given by

$$\mathbf{E}_{v,travel}^{TM} = -\frac{\tilde{k}}{2\sqrt{2\pi}} \int_{-\tilde{k}}^{\tilde{k}} \mathrm{d}k_x \frac{e^{ik_x x}}{\sqrt{2\pi}} \frac{1}{k_y} e^{ik_y(d+h/2)} \big(\cos(k_y y) - i\sin(k_y y) \big),$$

$$-\mathbf{E}_{v,travel}^{TM*} = \frac{\tilde{k}}{2\sqrt{2\pi}} \int_{-\tilde{k}}^{\tilde{k}} \mathrm{d}k_x \frac{e^{ik_x x}}{\sqrt{2\pi}} \frac{1}{k_y} e^{-ik_y(d+h/2)} \big(\cos(k_y y) + i\sin(k_y y) \big).$$

While $\mathrm{Asym}\mathbb{G}$ is technically semi-definite, the incident field $\mathbf{E}_{v,travel}$ is contained completely in the span of the non-singular eigenvectors $q_{cos}(k_y)$, $q_{sin}(k_y)$. Thus we can write down $\mathrm{Asym}\mathbb{G}^{-1} \cdot \mathbf{E}_{v,travel}$ as

$$\mathrm{Asym}\mathbb{G}^{TM-1} \cdot \mathbf{E}_{v,travel}^{TM} = -\frac{\tilde{k}}{2\sqrt{2\pi}} \int_{-\tilde{k}}^{\tilde{k}} \mathrm{d}k_x \frac{e^{ik_x x}}{\sqrt{2\pi}} \frac{1}{k_y} e^{ik_y(d+h/2)} \big(\rho_{cos}^{-1} \cos(k_y y) - i\rho_{sin}^{-1} \sin(k_y y) \big) \tag{S70}$$

which can be formally interpreted as a pseudo-inverse or the inverse restricted to the relevant non-singular subspace. Thus we have

$$\begin{aligned}
\big\langle \mathbf{E}_{v,travel}^{TM} \big| \, \mathrm{Asym}\mathbb{G}^{TM-1} \, \big| \mathbf{E}_{v,travel}^{TM} \big\rangle &= \frac{\tilde{k}^2}{8\pi} \int_{-\tilde{k}}^{\tilde{k}} \frac{1}{k_y^2} \left(\frac{a_{cos}^2}{\rho_{cos}} + \frac{a_{sin}^2}{\rho_{sin}} \right) \mathrm{d}k_x \\
&= \frac{\tilde{k}^2}{8\pi} \int_{-\tilde{k}}^{\tilde{k}} \frac{4}{\tilde{k}^2 k_y} \,\mathrm{d}k_x \\
&= \frac{1}{2\pi} \int_{-\tilde{k}}^{\tilde{k}} \frac{1}{\sqrt{\tilde{k}^2 - k_x^2}} \,\mathrm{d}k_x \\
&= \frac{1}{2}.
\end{aligned} \tag{S71}$$

A similar calculation yields

$$\left\langle -\mathbf{E}^*_{v,travel}\right| \text{Asym}\mathbb{G}^{-1} \left|\mathbf{E}_{v,travel}\right\rangle = \frac{\tilde{k}^2}{8\pi} \int_{-\tilde{k}}^{\tilde{k}} \frac{e^{ik_y(2d+h)}}{k_y^2} \left(\frac{a_{cos}^2}{\rho_{cos}} - \frac{a_{sin}^2}{\rho_{sin}} \right) \mathrm{d}k_x$$
$$= 0. \tag{S72}$$

This gives the traveling wave contribution to the material-independent LDOS enhancement limits

$$\rho_{sca}^{TM} = \frac{1}{4} \text{Re}\left\{ \left\langle -\mathbf{E}^{TM*}_{v,travel}\right| (\text{Asym}\mathbb{G}^{TM})^{-1} \left|\mathbf{E}^{TM}_{v,travel}\right\rangle \right\} + \frac{\tilde{k}}{4} \left\langle \mathbf{E}^{TM}_{v,travel}\right| (\text{Asym}\mathbb{G}^{TM})^{-1} \left|\mathbf{E}^{TM}_{v,travel}\right\rangle$$
$$= \frac{\tilde{k}}{8}. \tag{S73}$$

Thus, if only the traveling waves (far field) are considered, $\rho_{sca}^{TM}/\rho_0^{TM}$ tends towards a constant value of 1 in the single frequency limit. This can indeed be observed in the numerics for narrow bandwidth and large separation d, where the near-field contribution has decayed exponentially to the point of being negligible. An analogous calculation for TE shows that there the ratio is 1/2.

12.1.2 Evanescent wave contribution

We evaluate the bandwidth scaling of the evanescent wave contribution for lossless materials ($\chi_i = 0$) and TM polarization. To do so, we choose the phase parameter $p = 1 + ip_i$ with the imaginary part $p_i \to 0$. As we shall see, this not only simplifies the asymptotic analysis for small bandwidth but also will lead to material independent bounds that are finite.

Given $\chi_i = 0$, we have the material factor $\lim_{p_i \to 0} \text{Im}\{p/\chi^*\} \to 0$. Furthermore, from (S57) we have

$$r_- \sim \sqrt{k_{yi}^2 - k_{yr}^2 - \frac{2k_{yr}k_{yi}(B_i k_{yi} - B_r k_{yr})}{B_r k_{yi} + B_i k_{yr}}} \qquad r_+ \sim \sqrt{\frac{\chi(B_r k_{yi} + B_i k_{yr})}{p_i}} \qquad p_i \to 0 \tag{S74}$$

so we see that in the limit $p_i \to 0$, r_- tends toward a finite value while r_+ diverges $\propto p_i^{-1/2}$.

To simplify the analysis, we can use the Cauchy-Schwartz relaxed (S49) to get

$$\rho_{sca}^{TM} \le \frac{|\tilde{\omega}|}{2} \left\langle \mathbf{E}_v^{TM} \middle| \text{Asym}(p\mathbb{U}^{TM})^{-1} \middle| \mathbf{E}_v^{TM} \right\rangle$$
$$= \frac{1}{8\pi} \int_0^\infty \text{Re}\left\{ \frac{\left|\tilde{k}\right|^3 e^{-2k_{yi}d}}{\left|k_y\right|^2} \left(\frac{R_+}{r_+ + ik_y^*} + \frac{R_-}{r_- + ik_y^*} \right) \right\} \mathrm{d}k_x. \tag{S75}$$

Now, treating r_+ as a large variable in the limit $p_i \to 0$, asymptotic analysis of (S59) and (S61) gives

$$\frac{R_+}{r_+ + ik_y^*} + \frac{R_-}{r_- + ik_y^*} \sim \frac{8k_{yi}|k_y|^2}{\text{Im}\{p/\chi^*\} r_+^2 \left[(r_- + k_{yi})^2 + k_{yr}^2 \right]} \qquad p_i \to 0 \tag{S76}$$

Since $\text{Im}\{p/\chi^*\} \propto p_i$ and $r_+^2 \propto p_i^{-1}$ in the limit p_i, we see that the entire integrand of (S75) tends toward a finite value

$$\rho_{sca}^{TM} \le \frac{1}{8\pi} \int_0^\infty \text{Re}\left\{ \left|\tilde{k}\right|^3 e^{-2k_{yi}d} \frac{8k_{yi}}{(B_r k_{yi} + B_i k_{yr}) \left[(r_- + k_{yi})^2 + k_{yr}^2 \right]} \right\} \mathrm{d}k_x, \tag{S77}$$

so the bound with $p = 1$ is well-defined. We can now consider the narrow bandwidth limit $\tilde{k}_i \to 0$ given $p = 1$. It has already been established that the traveling wave contribution from the $k_x < \tilde{k}_r$ part of the integrand is a constant in the narrow bandwidth limit. In the evanescent region $kx > \tilde{k}_r$, to lowest order in $\tilde{k}_i$ we have

$$k_{yi} \sim \sqrt{k_x^2 - \tilde{k}_r^{\,2}} \quad k_{yr} \sim \frac{\tilde{k}_r}{k_{yi}} \cdot \tilde{k}_i \qquad \tilde{k}_i \to 0 \tag{S78a}$$

$$B_i \sim -\frac{\tilde{k}_r^2}{k_{yi}} \quad B_r \sim \left(\frac{\tilde{k}_r^3}{k_{yi}^3} + \frac{2\tilde{k}_r}{k_{yi}} \right) \cdot \tilde{k}_i \qquad \tilde{k}_i \to 0 \tag{S78b}$$

$$r_-(p = 1) \sim \sqrt{\tilde{k}_r^2 + k_{yi}^2} \qquad \tilde{k}_i \to 0 \tag{S78c}$$

All of these combine together to give

$$\mathrm{Re}\left\{ \left|\tilde{k}\right|^3 e^{-2k_{yi}d} \frac{8k_{yi}}{(B_r k_{yi} + B_i k_{yr}) \left[(r_- + k_{yi})^2 + k_{yr}^2 \right]} \right\} \propto \tilde{k}_i^{-1} \qquad kx > \tilde{k}_r, \tilde{k}_i \to 0 \tag{S79}$$

and we see that the evanescent wave contribution gives the inverse bandwidth scaling for lossless materials, as seen in the main text.

12.2 TM material Scaling

From section 12.1.2 we see that given lossless χ the bound with $p = 1$ is finite and well-defined, amounting to

$$\rho_{sca}^{TM} \leq \frac{\tilde{\omega}}{2} \left\langle \mathbf{E}_v^{TM} \middle| \mathrm{Asym}\mathbb{G}^{TM-1} \middle| \mathbf{E}_v^{TM} \right\rangle, \tag{S80}$$

which is a bound independent of χ, and thus therefore applies to all lossless χ. The next step is to show that (S80) is also a bound for lossy χ with $\mathrm{Im}(\chi) > 0$. We can see this by again setting $p = 1$ in (S48) and applying the Cauchy-Schwartz inequality to arrive at a bound for lossy χ:

$$\rho_{sca}^{TM} \leq \frac{|\tilde{\omega}|}{2} \left\langle \mathbf{E}_v^{TM} \middle| \left(\frac{\chi_i}{|\chi|^2} + \mathrm{Asym}\mathbb{G}^{TM} \right)^{-1} \middle| \mathbf{E}_v^{TM} \right\rangle. \tag{S81}$$

Now as operators we have $\frac{\chi_i}{|\chi|^2} \succ 0$ and $\mathrm{Asym}\mathbb{G}^{TM} \succeq 0$, so $\left\langle \mathbf{E}_v^{TM} \middle| \left(\frac{\chi_i}{|\chi|^2} + \mathrm{Asym}\mathbb{G}^{TM} \right)^{-1} \middle| \mathbf{E}_v^{TM} \right\rangle < \left\langle \mathbf{E}_v^{TM} \middle| \mathrm{Asym}\mathbb{G}^{TM-1} \middle| \mathbf{E}_v^{TM} \right\rangle$ and it is clear that the material independent bound (S80) while derived assuming $\chi_i = 0$ applies to general lossy χ. Thus in general as $|\chi| \to \infty$, the TM halfspace LDOS limits do not diverge, but are always bounded by (S80).

12.3 TM separation scaling

In this section we investigate the scaling of the TM half-space bounds with the vacuum separation d as $d \to 0$. The k_x integral in (S75) converges for finite d due to the exponential decay factor $e^{-2k_{yi}d}$: as $k_x \to \infty$, $k_{yi} \approx k_x \to \infty$. The scaling of (S75) with d thus depends on the k_x scaling of the rest of the integrand as $k_x \to \infty$.

12.3.1 Finite χ

Given finite χ, we can always select a complex phase p such that $\mathrm{Im}\{p/\chi^*\} > 0$. Now from (S43b) and (S78a) it is clear that $\lim_{k_x\to\infty} \mathrm{Asym}(p\mathbb{U}^{TM})_{k_x} = \mathrm{Im}\{p/\chi^*\}$, since $\lim_{k_x\to\infty} \tilde{k}^2/k_y = 0$ and $\lim_{k_x\to\infty} e^{ik_y|y-y'|} \to 0$ for $y \neq y'$ (if $y = y'$ then $e^{ik_y|y-y'|} = 1$ but this is a finite non-zero value of the integral kernel with support over a set of measure 0 and the integral operator as a whole still goes to 0).

The k_x integrand of $\left\langle \mathbf{E}_v^{TM} \middle| \mathrm{Asym}(p\mathbb{U}^{TM})^{-1} \middle| \mathbf{E}_v^{TM} \right\rangle$ given in (S44) for large k_x is then approximately

$$\frac{e^{-2k_x d}}{k_x^2} \mathrm{Im}\{p/\chi^*\}^{-1} \int_0^\infty e^{-2k_x y}\, \mathrm{d}y \propto \frac{e^{-2k_x d}}{k_x^3}.$$

Even at exactly $d = 0$, the integrand scales $\propto k_x^{-3}$ as $k_x \to \infty$ and the integral converges. Thus for finite χ the half-space LDOS bounds approach a finite constant as $d \to 0$.

12.3.2 Material independent bounds

For the material independent bound given in (S80), the k_x integrand is given by (S77) and (S78). In the narrow-bandwidth, large k_x limit the integrand

$$\mathrm{Re}\left\{ \left|\tilde{k}\right|^3 e^{-2k_{yi}d} \frac{8k_{yi}}{(B_r k_{yi} + B_i k_{yr}) \left[(r_- + k_{yi})^2 + k_{yr}^2\right]} \right\} \sim \frac{\left|\tilde{k}\right|^3}{k_r} \frac{1}{k_i} \frac{e^{-2k_x d}}{k_x} \qquad k_i \to 0, k_x \to \infty. \tag{S82}$$

Thus the material independent bound scaling as $d \to 0$ can be evaluated as

$$\begin{aligned} \rho_{sca,max}^{TM} &\sim \frac{1}{8\pi} \frac{\left|\tilde{k}\right|^3}{k_r} \frac{1}{k_i} \int_{\beta\tilde{k}_r}^\infty \frac{e^{-2k_x d}}{k_x} \mathrm{d}k_x \\ &= \frac{1}{8\pi} \frac{\left|\tilde{k}\right|^3}{k_r} \frac{1}{k_i} E_1(2\beta\tilde{k}_r d) \\ &\sim \frac{1}{8\pi} \frac{\left|\tilde{k}\right|^3}{k_r} \frac{1}{k_i} \ln(\lambda_0/d) \end{aligned} \tag{S83}$$

where we have selected a constant $\beta \gg 1$ such that the large k_x approximations hold; as seen in (S83) the exact value of β is irrelevant to the leading order asymptotic of $\ln(1/d)$. Note that this is a different scaling than the finite χ bounds in the previous subsection. A consequence of the material independent bounds diverging whilst fixed material bounds saturating as $d \to 0$ is that smaller d increases the relative advantage of large materials, as seen in Fig. (2b) in the main text.

12.4 TE separation scaling

The prior sections on the asymptotics of the TM case have demonstrated scaling of the bounds with the inverse of the bandwidth (for lossless materials) and saturation with increasing material susceptibility; the TE bounds share these scaling characteristics so for the sake of simplicity we will not do a detailed TE asymptotic analysis.

However, there is a difference in the $d \to 0$ scaling between the TM and TE results, which can be understood by comparing the $k_x \to \infty$ characteristics of $\mathbf{E}^{TM}_{v,k_x}$ and $\mathbf{E}^{TEy}_{v,k_x}$. For TE, we have

$$\begin{aligned}
\rho^{TEy}_{sca} &\leq 2 \int_0^\infty \left\langle \mathbf{E}^{TEy}_{v,k_x} \middle| \mathrm{Asym}(p\mathbb{U}^{TE}_{k_x})^{-1} \middle| \mathbf{E}^{TEy}_{v,k_x} \right\rangle \mathrm{d}k_x \\
&= 2 \int_0^\infty \left\langle \mathbf{E}^{TEy}_{v,k_x} \middle| (\mathrm{Im}\{p/\chi^*\} + \mathrm{Asym}(p^*\mathbb{G}^{TE}_{k_x}))^{-1} \middle| \mathbf{E}^{TEy}_{v,k_x} \right\rangle \mathrm{d}k_x \\
&< 2 \int_0^\infty \mathrm{Im}\{p/\chi^*\}^{-1} \| \mathbf{E}^{TEy}_{v,k_x} \|^2 \, \mathrm{d}k_x \\
&= \frac{1}{4\pi \left|\tilde{k}\right|^2} \int_0^\infty \frac{e^{-2k_{yi}d}}{2k_{yi}} \left(k_x^2 + \frac{k_x^4}{|k_y|^2} \right) \mathrm{d}k_x.
\end{aligned}$$

In the $k_x \to \infty$ limit, the integrand $\sim k_x e^{-2k_x d}$, so similar to (12.3.1), for the $d \to 0$ asymptotics we have

$$\begin{aligned}
\rho^{TEy}_{sca} &\lesssim \frac{1}{4\pi \left|\tilde{k}\right|^2} \int_{\beta \tilde{k}_r}^\infty k_x e^{-2k_x d} \, \mathrm{d}k_x \qquad d \to 0 \\
&= \frac{1}{d^2} \frac{1}{4\pi \left|\tilde{k}\right|^2} \cdot \frac{1}{4} e^{-2\beta \tilde{k}_r d} (2\beta \tilde{k}_r d + 1) \qquad d \to 0 \\
&\propto \frac{1}{d^2} \qquad d \to 0
\end{aligned} \tag{S84}$$

and we recover the $1/d^2$ scaling of the TE bounds seen in the main text.

12.4.1 3D separation scaling

For a point dipole near a 3D half-space design region, a similar analysis would yield $1/d^3$ scaling of the LDOS bounds. Due to the extra spatial dimension as compared with the TE case, the 1D k_x integral becomes a 2D $k_\parallel$ integral. For simplicity, suppose that the dipole is aligned with the normal of the design region surface, giving the problem cylindrical symmetry. The spatial integration kernel is then $2\pi \int_0^\infty k_\parallel (\cdot) \mathrm{d}k_\parallel$, with the bound integral analogous to (S84) having an extra $k_\parallel$ factor in the integrand. This will lead to $\propto 1/d^3$ scaling, as seen in [5].

13 Comparison to passivity bounds

The main prior work concerning finite bandwidth LDOS limits is [5], where an bound based on passivity constraints was derived:

$$\rho_{sca} \leq \frac{|\tilde{\omega}|}{2} f(\tilde{\omega}, \chi) \langle \mathbf{E}_v | \mathbf{E}_v \rangle \tag{S85a}$$

with the material figure of merit

$$f(\tilde{\omega}, \chi) = \frac{|\tilde{\omega}\chi|^2 + |\tilde{\omega}\chi| \Delta\tilde{\omega}}{|\tilde{\omega}| \, \mathrm{Im}(\tilde{\omega}(1+\chi))}. \tag{S85b}$$

Note that compared to (22) in [5], we have dropped the electrostatic contribution (as in the main text), and there is an additional factor of $\pi|\tilde{\omega}|^2$ due to differences in definitions (the π factor is a matter of convention; the $|\tilde{\omega}|^2$ factor comes from $\mathbf{E}_v$ in our work coming from a unit amplitude dipolar current source instead of a unit dipole moment). Compared to the material bound given by (16) in the main text, (S85) essentially corresponds to a particular value of p. For example, given a lossless χ and narrow bandwidth $\Delta\omega \ll |\tilde{\omega}|$, we have

$$f(\tilde{\omega}, \chi) \approx \frac{|\tilde{\omega}|}{\Delta\omega} \frac{\chi^2}{1+\chi}.$$

Now if we select a phase rotation p such that $p_r = 1$,

$$p_i = \frac{\chi+1}{\chi} \frac{\Delta\omega}{|\tilde{\omega}|}, \tag{S86}$$

the material bound is

$$\frac{|\tilde{\omega}|}{2} \operatorname{Im}\{p/\chi^*\}^{-1} \langle \mathbf{E}_v | \mathbf{E}_v \rangle = \frac{|\tilde{\omega}|}{2} \frac{|\tilde{\omega}|}{\Delta\omega} \frac{\chi^2}{1+\chi} \langle \mathbf{E}_v | \mathbf{E}_v \rangle = \frac{|\tilde{\omega}|}{2} f(\tilde{\omega}, \chi) \langle \mathbf{E}_v | \mathbf{E}_v \rangle$$

which is exactly equivalent to the passivity bound. Checking (S39) it is clear that with such a phase rotation p, $\operatorname{Asym}(p\mathbb{U})$ remains positive definite.

13.1 Bandwidth scaling of the passivity bounds

The passivity bounds (S85) have increased bandwidth scaling given an infinite halfspace design region coming from traveling wave contributions to $\langle \mathbf{E}_v | \mathbf{E}_v \rangle$. For example, given TM polarization we have

$$\begin{aligned} \left\langle \mathbf{E}_v^{TM} \middle| \mathbf{E}_v^{TM} \right\rangle &= \frac{\left|\tilde{k}\right|^2}{8\pi} \int_{-\infty}^{\infty} \frac{e^{-2k_{yi}d}}{|k_y|^2} \int_0^{\infty} e^{-2k_{yi}y} \mathrm{d}y \, \mathrm{d}k_x \\ &= \frac{\left|\tilde{k}\right|^2}{8\pi} \int_{-\infty}^{\infty} \frac{e^{-2k_{yi}d}}{2|k_y|^2 k_{yi}} \mathrm{d}k_x. \end{aligned}$$

Now for traveling waves $|k_x| < \tilde{k}_r$, it can easily be shown from $k_y = \sqrt{\tilde{k}^2 - k_x^2}$ that $k_{yi} \propto \tilde{k}_i$, leading to $\left\langle \mathbf{E}_v^{TM} \middle| \mathbf{E}_v^{TM} \right\rangle \propto 1/\tilde{k}_i \propto 1/\Delta\omega$ (this analysis also applies to TE polarization). For lossless dielectrics, $f(\tilde{\omega}, \chi) \approx \frac{|\tilde{\omega}|}{\Delta\omega}$, giving the passivity bounds an overall $1/\Delta\omega^2$ scaling, as compared to the $1/\Delta\omega$ scaling for the full bounds. For lossy materials $f(\tilde{\omega}, \chi) \approx \frac{|\chi|^2}{\chi_i}$, giving the passivity bounds an overal $1/\Delta\omega$ scaling, as compared to the $1/\Delta\omega^4$ scaling of the full bounds seen in Fig (3) of the main text.

References

[1] Tyler W Hughes, Ian AD Williamson, Momchil Minkov, and Shanhui Fan. Forward-mode differentiation of Maxwell's equations. *ACS Photonics*, 6(11):3010–3016, 2019.

[2] S. G. Johnson et al. *The NLopt Nonlinear Optimization Package (Version 2.6.2)*. August 2019.

[3] Sean Molesky, Pengning Chao, and Alejandro W. Rodriguez. Hierarchical mean-field $\mathbb{T}$ operator bounds on electromagnetic scattering: Upper bounds on near-field radiative Purcell enhancement. *Physical Review Research*, 2(4):043398, December 2020.

[4] Walter Rudin. *Real and Complex Analysis*. McGraw-Hill Education, New York, 2006.

[5] Hyungki Shim, Lingling Fan, Steven G. Johnson, and Owen D. Miller. Fundamental Limits to Near-Field Optical Response over Any Bandwidth. *Physical Review X*, 9(1):011043, March 2019.

[6] Leung Tsang, Jin Au Kong, and Kung-Hau Ding. *Scattering of Electromagnetic Waves: Theories and Applications*, volume 27. John Wiley & Sons, 2004.

[7] Aaron Welters, Yehuda Avniel, and Steven G. Johnson. Speed-of-light limitations in passive linear media. *Physical Review A*, 90(2):023847, August 2014.
